# Genome-wide study of salivary microRNAs as potential noninvasive biomarkers for detection of nasopharyngeal carcinoma

**DOI:** 10.1186/s12885-019-6037-y

**Published:** 2019-08-28

**Authors:** Lirong Wu, Kexiao Zheng, Cheng Yan, Xuan Pan, Yatian Liu, Juying Liu, Feijiang Wang, Wenjie Guo, Xia He, Jiong Li, Ye Shen

**Affiliations:** 1 Department of Radiation Oncology, Jiangsu Cancer Hospital & Jiangsu Institute of Cancer Research & The Affiliated Cancer Hospital of Nanjing Medical University, Nanjing, 210009 China; 2 Nano-Bio-Chem Centre, Suzhou Institute of Nano-Tech and Nano-Bionics, Chinese Academy of Sciences, Suzhou, 215123 China; 3 Department of Oncology, Jiangsu Cancer Hospital & Jiangsu Institute of Cancer Research & The Affiliated Cancer Hospital of Nanjing Medical University, Nanjing, 210009 China

**Keywords:** Nasopharyngeal carcinoma, Biomarkers, MicroRNA, Saliva

## Abstract

**Background:**

Recent studies reported that blood-based microRNAs (miRNAs) could detect cancers and predict prognosis have opened a new field of utilizing circulating miRNAs as cancer biomarkers. In this pilot study, we conducted for the first time, to our knowledge, the evaluation of the applicability of salivary miRNAs as novel biomarkers for nasopharyngeal carcinoma (NPC) detection.

**Methods:**

Microarray miRNA expression profiling was performed on saliva samples from 22 newly diagnosed NPC patients and 25 healthy controls, and 12 significantly down-regulated miRNAs were selected for quantitative real-time-PCR (qRT-PCR) validation and further analysis. Their target genes enriched by gene ontology and pathway analysis were used to construct regulatory and interaction networks. The receiver operating characteristic analyses (ROC) and logistic regression were calculated to assess discriminatory accuracy.

**Results:**

Twelve dysregulated miRNAs screened by microarray that showed the same expression patterns with qRT-PCR analysis. Through bioinformatics analysis, the most prominent hub gene probably regulated by the 12 down-regulated miRNAs is found to be TP53. The ROC including the 12 miRNAs separated NPC patients from healthy controls with very high accuracy (areas under the receiver operating characteristic curve [AUC] = 0.999, sensitivity = 100.00%, specificity = 96.00%). Furthermore, if only six significantly dysregulated miRNAs were selected for the ROC analysis, the accuracy is still impressive (AUC = 0.941, sensitivity = 95.45%, specificity = 80.00%).

**Conclusions:**

This study highlights the potential for salivary miRNAs as biomarkers for the detection of NPC. Meanwhile, differentially expressed miRNAs in saliva might play critical roles in NPC by regulating their target genes, which associated with some significant pathways, such as p53 signaling pathway.

**Electronic supplementary material:**

The online version of this article (10.1186/s12885-019-6037-y) contains supplementary material, which is available to authorized users.

## Background

Nasopharyngeal carcinoma (NPC) is a cancer arising from the nasopharynx epithelium, and quite rare in most regions of the world, with incidence rates below 1 per 100,000 person-years. However, it is rather prevalent in southern China, southeast Asia and northern Africa [1]. In terms of demographic trends, men are three times more likely to develop the disease than women, and the peak age is between 50 and 60 years old [2]. NPC is highly associated with Epstein-Barr virus (EBV) infection, genetic susceptibility, smoking and drinking, and environmental factors are also risk factors [3]. For early-stage NPC, radiotherapy is often curative, while patients diagnosed at advanced stages always have poorer outcomes. Therefore, early detection is essential to reduce the burden of NPC.

With the progress of molecular pathogenesis research related to NPC, a number of biomarkers associated with diagnosis and prognosis have been reported, including EBV DNA, circulating microRNAs (miRNAs), cytokines and methylated genes [3–6]. Nevertheless, detection of these molecules generally depends on invasive sample collection such as fresh or formalin fixed paraffin-embedded (FFPE) tissue, plasma or serum, which causes discomfort to patients. Previous studies reported bodily fluid type-specific molecules probably have functional roles associated with the surrounding tissues [7]. Saliva, an easy-to-access bodily fluid near nasopharyngeal tissue, is a promising non-invasive sample for the detection of NPC biomarkers. Due to the exchange of substances between blood and tissue during circulation, and the extensive blood supply in salivary glands, the molecules that present in tissue and plasma are probably also present in saliva [8]. Therefore saliva molecules may be used to detect human systemic disease, especially the diseases of tissues near the salivary gland.

miRNAs are 19–23 nucleotide-long, single-stranded small RNA molecules which regulate the production of proteins from messenger RNA (mRNA) [9]. Many studies have reported that aberrant expression of miRNAs is closely associated with tumorigenicity, including NPC [10–12]. For example, suppression of miR-29c subsequently increases the migration and invasion of NPC cells through up-regulation of its targeting mRNAs that encode extracellular matrix proteins (collagens 3A1, 4A1, 15A1, and laminin γ1) [10]. Overexpression of miR-378 in NPC tissues downregulates the expression of (TOB2, a potential tumor suppressor) and dramatically promotes cell proliferation, colony formation, migration, and invasion in vitro [11]. Moreover, proposed targets of miR-26a, miR-98, miR-155, miR-200a/b, miR-205 and miR-216b regulate many important processes such as the epithelial-to-mesenchymal transition (EMT) as well as signaling pathways including Notch, phosphatidylinositol-4,5-bisphosphate 3-kinase (PI3K)-Akt, and mitogen-activated protein kinase (MAPK) which are involved in nasopharyngeal tumorigenesis [12].

It is not surprising that miRNAs, a pivotal class of epigenetic regulators, are deemed to possess discriminatory power for many diseases, including cancer [13]. Yet it is truly remarkable that miRNAs can serve as valuable biomarkers because of their widespread presence in the body. Recent studies revealed that miRNAs are not only found in tissues, but also in plasma, serum, saliva, urine and other bodily fluids, and exist in a stable extracellular form [7, 14]. Specifically for salivary miRNAs, several new research shows that they can be considered as promising biomarkers for the detection of esophageal cancer [15, 16]. However, miRNA expression in the saliva of NPC patients has not yet been reported. We presented in this pilot study, to our knowledge for the first time, the evaluation of the potential of a salivary miRNA panel as non-invasive diagnostic NPC biomarkers. The panel is screened by comparing the expression profile of salivary miRNAs in newly diagnosed and untreated NPC patients to that of the healthy donors.

## Methods

### Patients and controls

Saliva samples from 22 newly diagnosed NPC patients and 25 healthy donors (discovery cohort) used for candidate miRNA biomarker screening and an additional collection of saliva samples from 8 NPC patients and 8 healthy donors (validation cohort) used for evaluation of the screened candidates were obtained from Nanjing Medical University Affiliated Cancer Hospital (Nanjing, China) between March 2015 and November 2016. All the patients had no malignant tumor history and had not undergone any therapeutic procedures (such as chemotherapy or radiotherapy) previously. Cancer staging was based on the 8th edition of the International Union against Cancer/American Joint Committee on Cancer (UICC/AJCC) system [17]. The specimen of saliva collected from healthy donors were confirmed not to have NPC or other inflammatory condition, and no history of any malignant diseases. The characteristics of NPC patients and healthy controls were summarized in Table 1. This research work was approved by the Research Ethics Committee of Jiangsu Cancer Hospital. All patients and healthy controls gave written informed consent for participation in this study.

**Table 1:** Characteristics of NPC patients and healthy controls

Characteristics	Discovery set (*n* = 47)			Validation set (*n* = 16)		
	NPC patients (*n* = 22)	healthy controls (*n* = 25)	*p* value	NPC patients (*n* = 8)	healthy controls (*n* = 8)	*p* value
Median age (y, range)	48 (25–68)	45 (25–65)	0.933	50 (20–69)	46 (26–61)	0.311
Gender			0.756			0.619
Male	14 (63.6%)	16 (64.0%)		6 (75%)	5 (62.5%)	
Female	8 (36.4%)	9 (36.0%)		2 (25%)	3 (37.5%)	
Smoking			0.713			0.642
Yes	10 (45.5%)	10 (40.0%)		4 (50%)	5 (62.5%)	
No	12 (54.5%)	15 (60.0%)		4 (50%)	3 (37.5%)	
Drinking			0.825			0.334
Yes	3 (13.6%)	4 (16.0%)		1 (12.5%)	0	
No	19 (86.4%)	21 (84.0%)		7 (87.5%)	8 (100%)	
Karnofsky performance score			0.129			0.334
≤ 80	2 (9.1%)	0		1 (12.5%)	0	
≥ 90	20 (90.9%)	25 (100/%)		7 (87.5%)	8 (100%)	
Pathology (WHO classification)						
Non-keratinizing (typeII-III)	22 (100.0%)			8 (100.0%)		
Pre-EBV DNA (copies/mL) Median (rang)	1,597,817 (0–59,200,000)	0	0.001	1,959,159 (0–2,860,000)	0	0.022
LDH (U/L) Median (range)	168 (102–684)	115 (101–298)	0.368	171 (116–339)	137 (102–274)	0.119
HGB(g/L) Median (range)	127 (81–169)	135 (121–168)	0.289	130 (93–172)	129 (117–148)	0.085
ALB(g/L) Median (range)	41.3 (30–55)	46 (37–56)	0.041	41 (34–56)	44 (38–55)	0.215
CRP (mg/L) Median (range)	2 (0–119.1)	1.2 (0–32)	0.025	8.1 (0–45.4)	2.3 (0–29)	0.063
T category ^a^						
T1	6 (27.3%)			1 (12.5%)		
T2	4 (18.2%)			1 (12.5%)		
T3	5 (22.7%)			3 (37.5%)		
T4	7 (31.8%)			3 (37.5%)		
N category ^a^						
N0	1 (4.5%)			0		
N1	6 (27.3%)			3 (37.5%)		
N2	12 (54.6%)			4 (50.0%)		
N3	3 (13.6%)			1 (12.5%)		
Overall stage ^a^						
I	1 (4.5%)			0		
II	5 (22.7%)			2 (25.0%)		
III	6 (27.3%)			5 (50.0%)		
IVA-B	10 (45.5%)			1 (12.5%)		

*Abbreviations*: *WHO* world health organization, *Pre-EBV DNA* pre-treatment plasma Epstein-Barr Virus DNA, *LDH* lactate dehydrogenase, *HGB* hemoglobin, *ALB* albumin, *CRP* C-reaction protein

^a^According to the 8th AJCC/UICC staging system

### Saliva collection

Unstimulated saliva was collected as reported previously with minor modification [18]. Briefly, participants were asked to refrain from eating, drinking and oral hygiene procedures for at least 2 h before the collection. Subjects rinsed their mouth with water before saliva collection to minimize contamination of the samples. Five minutes later, the participants were asked to sit upright and spit into a 50-ml centrifuge tube kept on ice. At least 4 ml saliva per subject was collected within 20 min. Samples were then centrifuged at 3000×g, 4 °C for 15 min to spin down exfoliated cells. Three microliter saliva supernatant was transferred to a new tube on ice. All saliva supernatant samples were stored at − 80 °C until used.

### RNA sample preparation

To extract total RNA from saliva samples, miRNeasy Serum/Plasma Kit (Qiagen, Hilden, Germany) and TRIzol-LS reagent (Ambion, Life Technologies, Carlsbad, CA) were used. In brief, 3 μl saliva supernatant per sample was lysed using 6 mL of TRIzol-LS reagent. After vortexing with 1.6 ml of chloroform, samples were then centrifuged at 12,000×g for 15 min at 4 °C. The aqueous phase was transferred to a new tube and 1.5 fold volume of ethanol was added. At last, the sample was applied to the column for RNA collection according to the miRNeasy serum/plasma handbook.

### miRNA profiling by SHUT miRNA array

The fluorescent microarray-based label-free profiling method termed Stacking-Hybridized Universal Tag (SHUT) assay containing probes for 2025 human miRNAs from Sanger miRBase 19.0 was used according to the conditions published previously [19]. Total RNA from each sample and 200 nM (final concentration) Cy3-labeled reporter molecule (Universal Tag, UT) were dissolved in the hybridization buffer. The array was hybridized at 44 °C for 48 h in a hybridization oven (Agilent 2545A). After hybridization, slides were scanned using a LuxScan 10 K Microarray Scanner (CapitalBio, Beijing, China) at constant power and PMT gain settings through a single-color channel (532 nm wavelength). Finally, raw data were collected with GenePix Pro 7.0 software package (Axon, CA).

Data analysis was carried out within the R statistical computing framework version 2.8.0. The signal after background subtraction was normalized using quantile normalization strategies. For further analysis, the values from four replicate spots for each miRNA were summarized as median signals and only those miRNAs that showed a signal greater than 300 in at least one of all samples were used for subsequent analyses. Differentially expressed miRNAs were selected using the paired *t*-test with the cut-off criteria of *P* < 0.05 and |fold change| > 1.5. In order to ensure the differentially expressed miRNAs were accurately identified, hierarchical clustering was employed to cluster samples and groups with similar miRNA profiles.

### Quantitative real-time-PCR (qRT-PCR)

The relative quantification of selected differentially expressed miRNAs was performed by qRT-PCR on a Roche LightCycler System (Roche Diagnostics, Rotkreuz, Switzerland). The miRNA specific primers were designed by Oligo 7.0 and listed in Additional file 1: Table S6. Briefly, extracted total RNA from 2 ml salivary samples (with 10 μl of 10 nM cel-miR-39-3p spiked-in) was polyadenylated by poly(A) polymerase (Ambion, Austin, TX) at 37 °C for 1 h in a 25 μl reaction mixture following the manufacturer’s instructions. Then, the RNAs were reverse transcribed into cDNAs using the PrimeScript™ RT reagent Kit (#RR037A, TaKaRa) with specific RT primers for miRNAs. Each reaction was performed in 10 μl volume containing 1 μl cDNA, 0.5 μl of each universal reverse primer and miRNA specific primer and 5 μl of 2× SYBR Premix Ex Taq™ II (#RR820A, TaKaRa) Mix. The spiked cel-miR-39-3p was used as an external control for normalization. Cycling conditions were as follows: 95 °C for 30 s, 95 °C for 5 s and 60 °C for 50 s, followed by 40 cycles. The relative expression level of miRNAs were calculated using the 2^-△△Ct^ method.

### Bioinformatics

The miRNA targets were predicted by at least two databases of the following usual prediction databases: TargetScan, miRanda and miRTarBase. The Gene Ontology (GO) functional and pathway enrichment analysis were conducted for the target genes using the Database for Annotation, Visualization and Integrated Discovery (DAVID, http://david.abcc.ncifcrf.gov/home.jsp) with the cut-off criterion of false discovery rate (FDR) < 0.05. The protein-protein interactions (PPI) for these target genes was revealed using the Search Tool for the Retrieval of interacting Genes database (STRING, http://string.embl.de/). The miRNA-target gene regulatory network and PPI network were visualized using Cytoscape (Version 3.1.1).

### Statistical analysis

The statistical analysis was performed by using the SPSS software version 22.0 (SPSS Inc. Chicago, IL, USA) and MedCalc software version 15.8 (MedCalc, Mariakerke, Belgium). The Mann-Whitney U test was used to determine the significance of different levels of miRNA expression. Logistic regression was used to combine some miRNAs to a score which is interpreted as a diagnostic marker for discrimination of cases and controls. Receiver operating characteristic (ROC) curves were plotted to determine the specificity and sensitivity of miRNA as a diagnostic biomarker.

## Results

### Screening of differentially expressed miRNAs and qRT-PCR validation

**Fig. 1:**
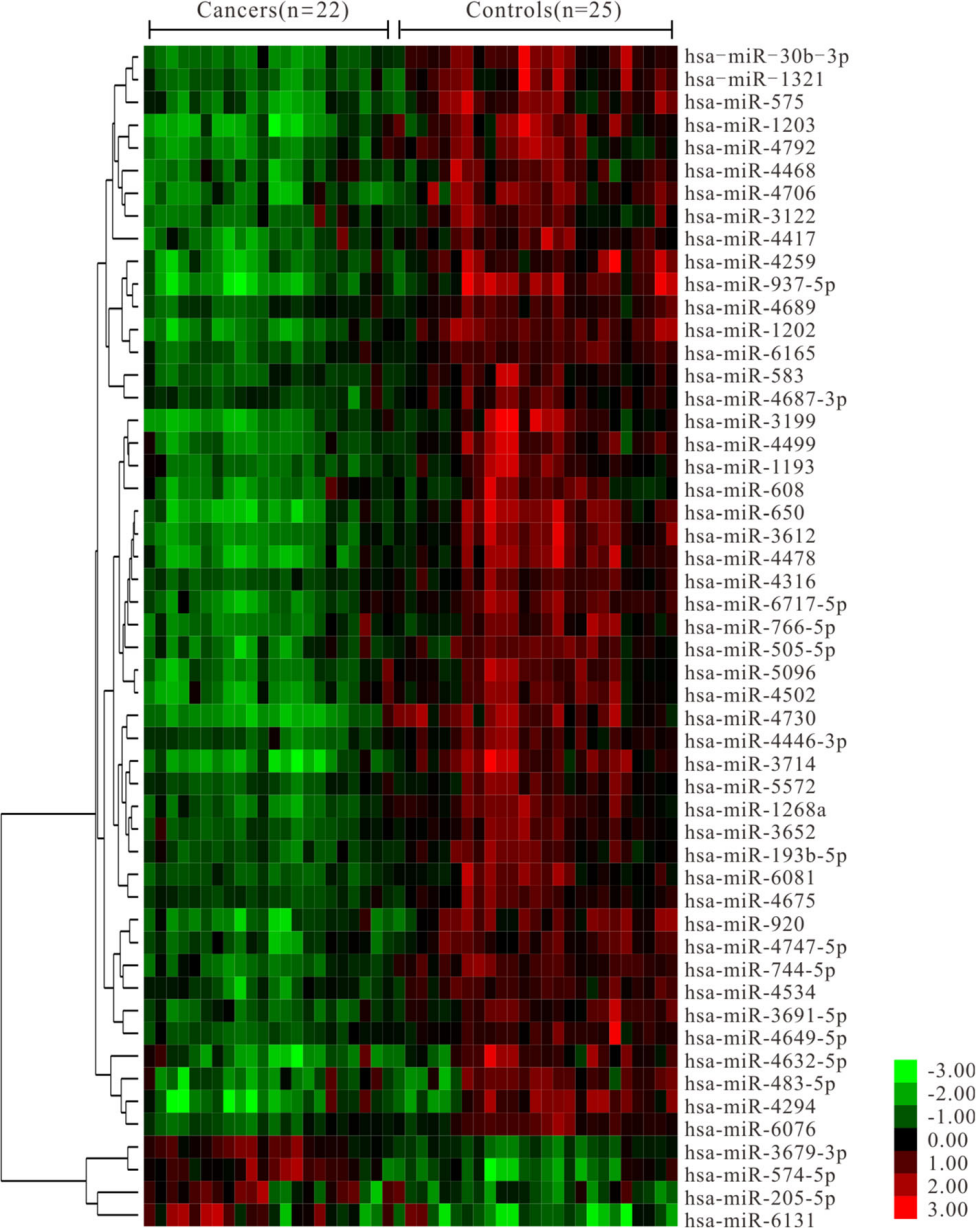
Hierarchical clustering heat map of the differentially expressed miRNAs. Each column corresponds to a single microarray, whereas each row indicates the expression profile of a single miRNA. Red and green represent high and low miRNAs expression, respectively. *P* < 0.05 and |fold change| > 1.5 were used as cutoff criteria

The differentially expressed miRNAs between NPC and healthy controls were screened using the high throughput microarray-based SHUT platform contained 2025 human miRNA probes. A total of 1105 miRNAs were detected in saliva samples (the probe signal value of these miRNAs was present in at least one sample). Of these miRNAs, 1064 were detected in NPC samples and 1013 in healthy control samples. Fifty-one miRNAs were aberrantly expressed in NPC saliva samples relative to healthy controls (Fig. 1). In detail, expression of salivary miR-3679-3p, miR-574-5p, miR-205-5p, and miR-6131 increased in NPC patients. Meanwhile, 47 miRNAs, including miR-30b-3p, miR-575, and miR-650, were down-regulated in NPC patients compared to healthy controls. Furthermore, hierarchical clustering analysis revealed the distinct expression of all differentially expressed miRNAs between the saliva samples of NPC patients and healthy controls.

**Fig. 2:**
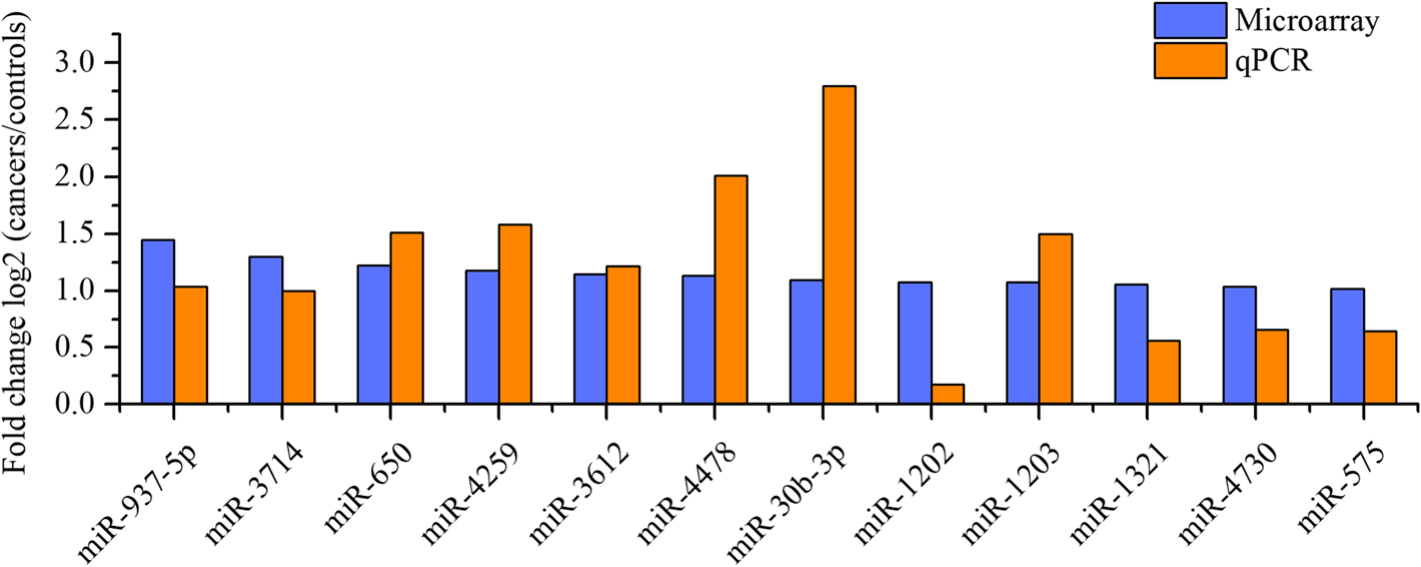
Validation of selected 12 miRNAs by qRT-PCR. Expression levels of miR-937-5p, miR-650, miR-3612, miR-4478, miR-4259, miR-3714, miR-4730, miR-1203, miR-30b-3p, miR-1321, miR-1202, and miR-575 in saliva were measured in 8 NPC patients and 8 controls

We selected 12 miRNAs (miR-937-5p, miR-650, miR-3612, miR-4478, miR-4259, miR-3714, miR-4730, miR-1203, miR-30b-3p, miR-1321, miR-1202 and miR-575) as the candidate miRNAs based on the higher cut-off values of *P* < 0.01 and |fold change| > 2 for further qRT-PCR validation (Additional file 1: Table S1). First, to confirm our findings, expression level of this panel in the same samples were quantified by qRT-PCR (Additional file 1: Figure S1), the results suggest that their change patterns were in accordance with the microarray analysis. Next, the expression levels of these candidate miRNAs were measured in an independent cohort of saliva from 8 NPC patients and 8 healthy controls. The qRT-PCR results revealed that all of the 12 miRNAs were down-regulated in NPC and showed the same expression patterns in microarray analysis (Fig. 2).

### Functional and pathway enrichment analysis

**Fig. 3:**
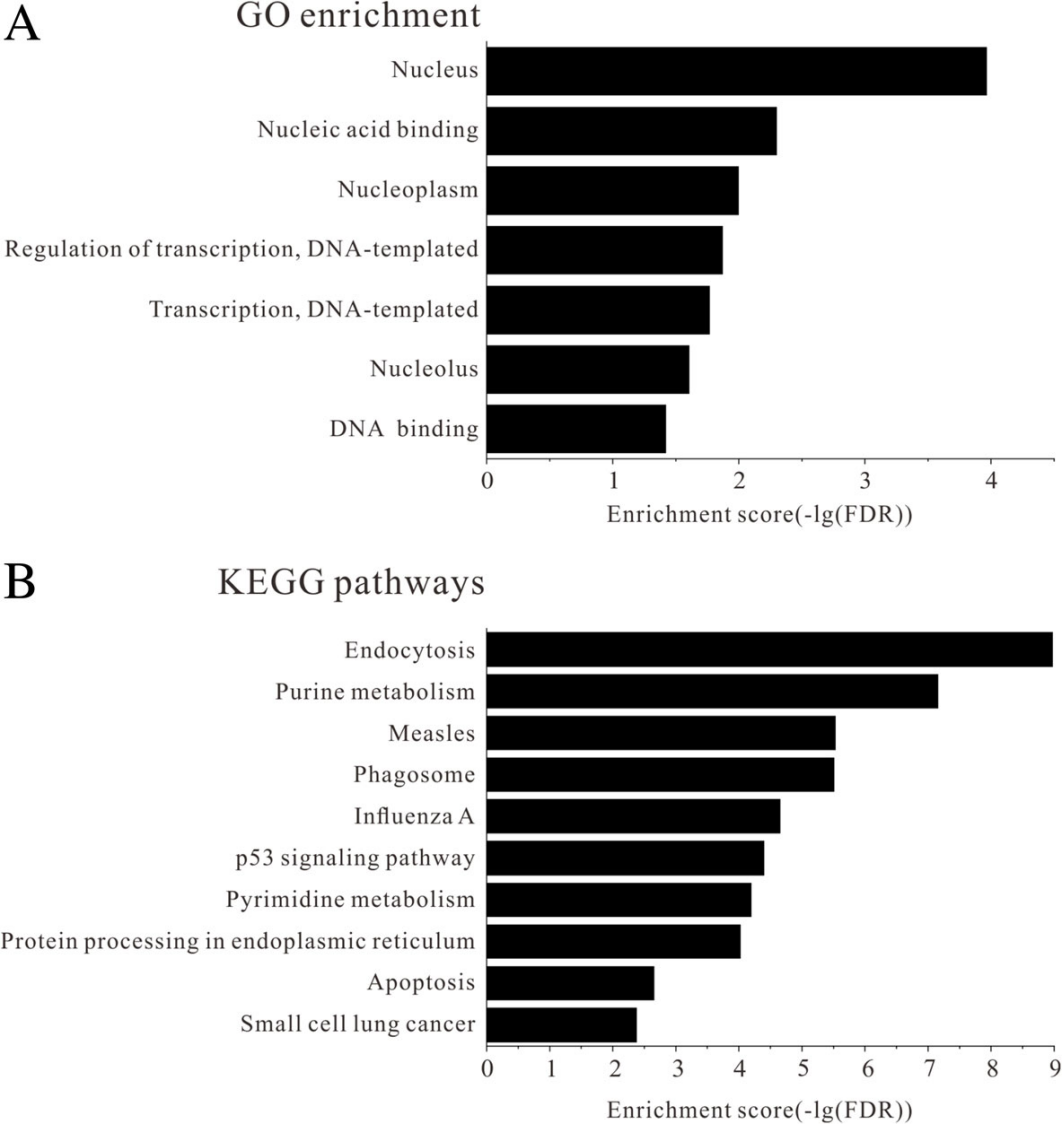
Enrichment analysis of GO function and pathways. **a** GO function enrichment analysis and **b** KEGG pathway enrichment analysis for predicted miRNAs targets different expressed between NPC patients and controls. Only the top ten pathways were shown. FDR < 0.05 was set as criteria for analysis

We selected 12 validated miRNAs for functional and pathway enrichment analysis. The predicted target genes for these miRNAs were listed in Additional file 1: Table S2. GO annotations indicate that these target genes were associated with biological characteristics like regulation of transcription (DNA-templated) (GO:0006355) and transcription (DNA-templated) (GO:0006351); molecular functions including nucleic acid binding (GO:0003676) and DNA binding (GO:0003677); and the gene products were primarily found in nucleus (GO:0005634), nucleoplasm (GO:0005654), and nucleolus (GO:0005730) (Fig. 3a, Additional file 1: Table S3). The top ten enriched pathways as revealed by Kyoto Encyclopedia of Genes and Genomes (KEGG) analysis, includes endocytosis (hsa04144), purine metabolism (hsa00230), and tumor protein 53 (p53) signaling pathway (hsa04115) (Fig. 3b, Additional file 1: Table S4). As these analyzed miRNAs were down-regulated in NPC samples, the expression level of target genes that enriched in GO terms and pathways were probably elevated in NPC patients since miRNAs were known as negative regulator of their target genes [20].

**Fig. 4:**
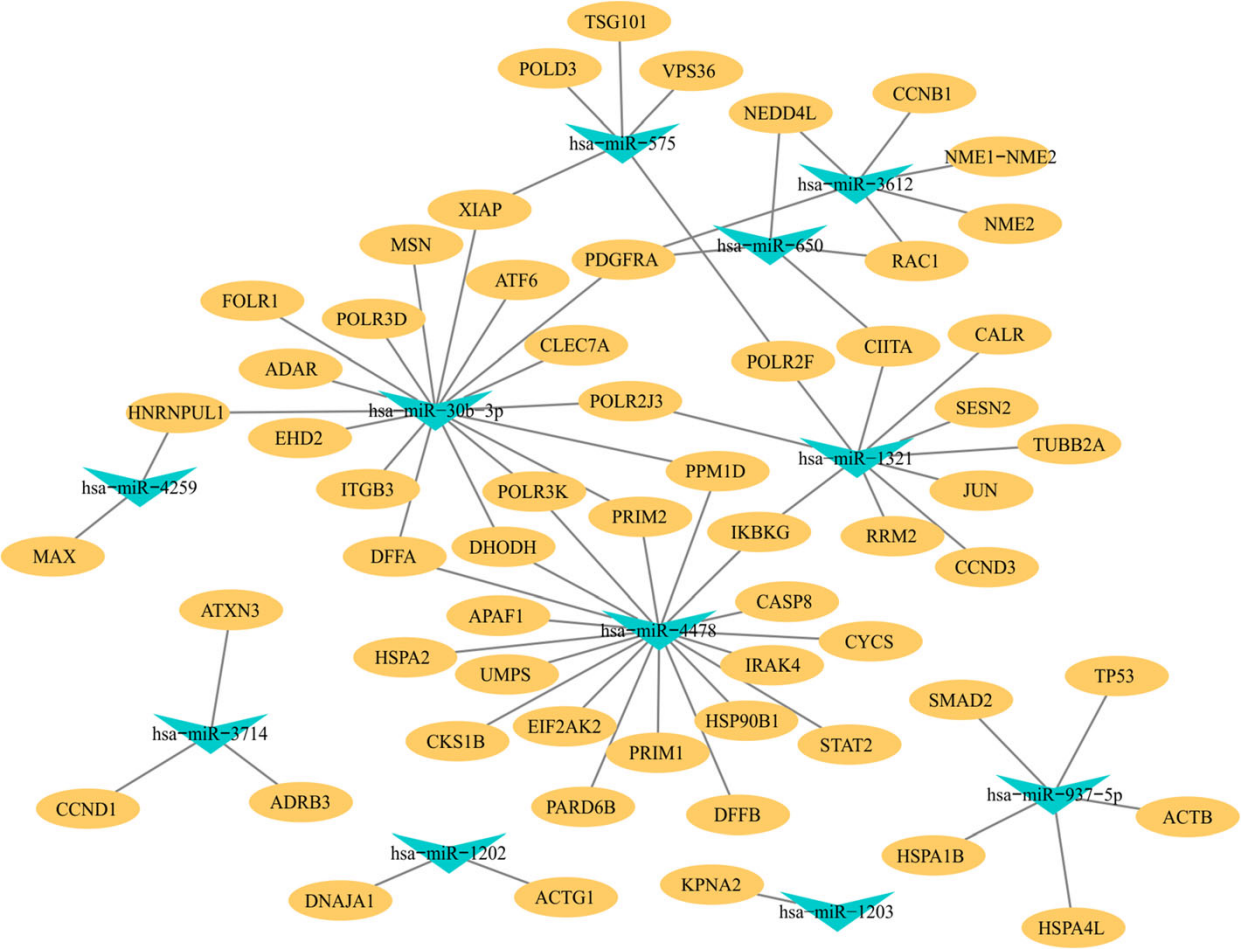
The regulatory network for differentially expressed miRNAs. The yellow nodes represent the target genes. The green triangles indicate differentially expressed miRNAs. The black lines show the potential regulatory relationships between miRNAs and genes

In the constructed regulatory network (Fig. 4), platelet-derived growth factor receptor alpha (PDGFRA) was simultaneously targeted by miR-3612, miR-650, and miR-30b-3p. Some other targets, such as Ras-related C3 botulinum toxin substrate 1 (RAC1), inhibitor of nuclear factor kappa B kinase subunit gamma (IKBKG), X-linked inhibitor of apoptosis protein (XIAP), and protein phosphatase, Mg^2+^/Mn^2+^ dependent 1D (PPM1D) were simultaneously regulated by two kinds of miRNAs. The target genes of miR-4730 were not involved in the enriched GO annotations and pathways and are therefore absent from the network.

**Fig. 5:**
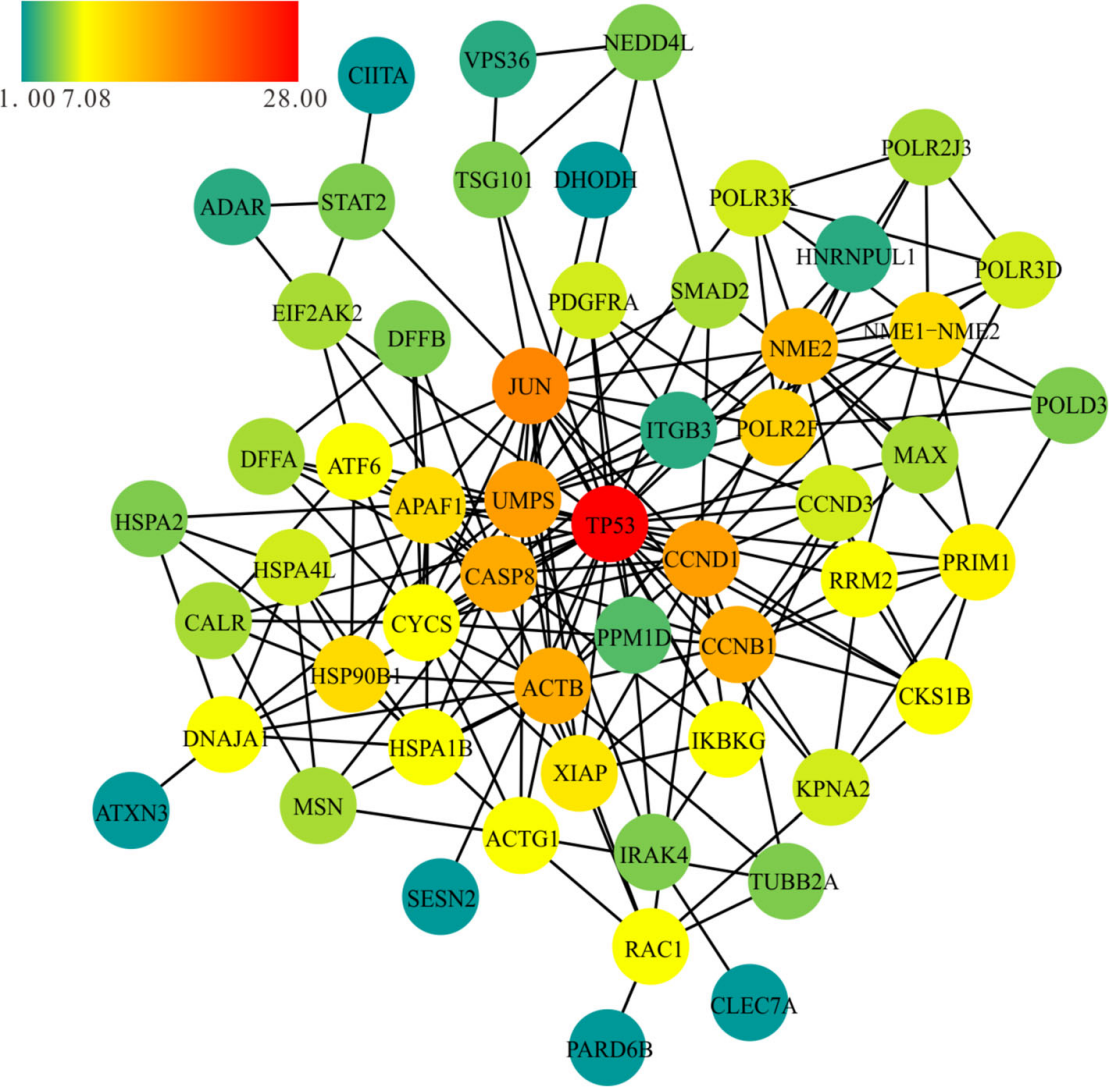
Protein-protein interaction (PPI) network for the predicted target genes of differentially expressed miRNAs in NPC. Colored bars indicate the degree of genes

In the PPI network predicted by STRING (Fig. 5), the most significant hub molecule is TP53 (which encodes the p53 protein). It had the highest degree centrality in the network. In addition, Jun proto-oncogene AP-1 transcription factor subunit (JUN) (degree = 17), uridine monophosphate synthetase (UMPS) (degree = 15), cyclin D1 (CCND1) (degree = 15), caspase 8 (CASP8) (degree = 14), cyclin B1 (CCNB1) (degree = 14), actin beta (ACTB) (degree = 14), cytochrome c somatic (CYCS) (degree = 14), and NME/NM23 nucleoside diphosphate kinase 2 (NME2) (degree = 13) are also significant hubs (Additional file 1: Table S5). The interaction confidence (benchmarked as the “combined score” by STRING) are list in Additional file 1: Table S7.

### Diagnostic utility of potential miRNA

**Fig. 6:**
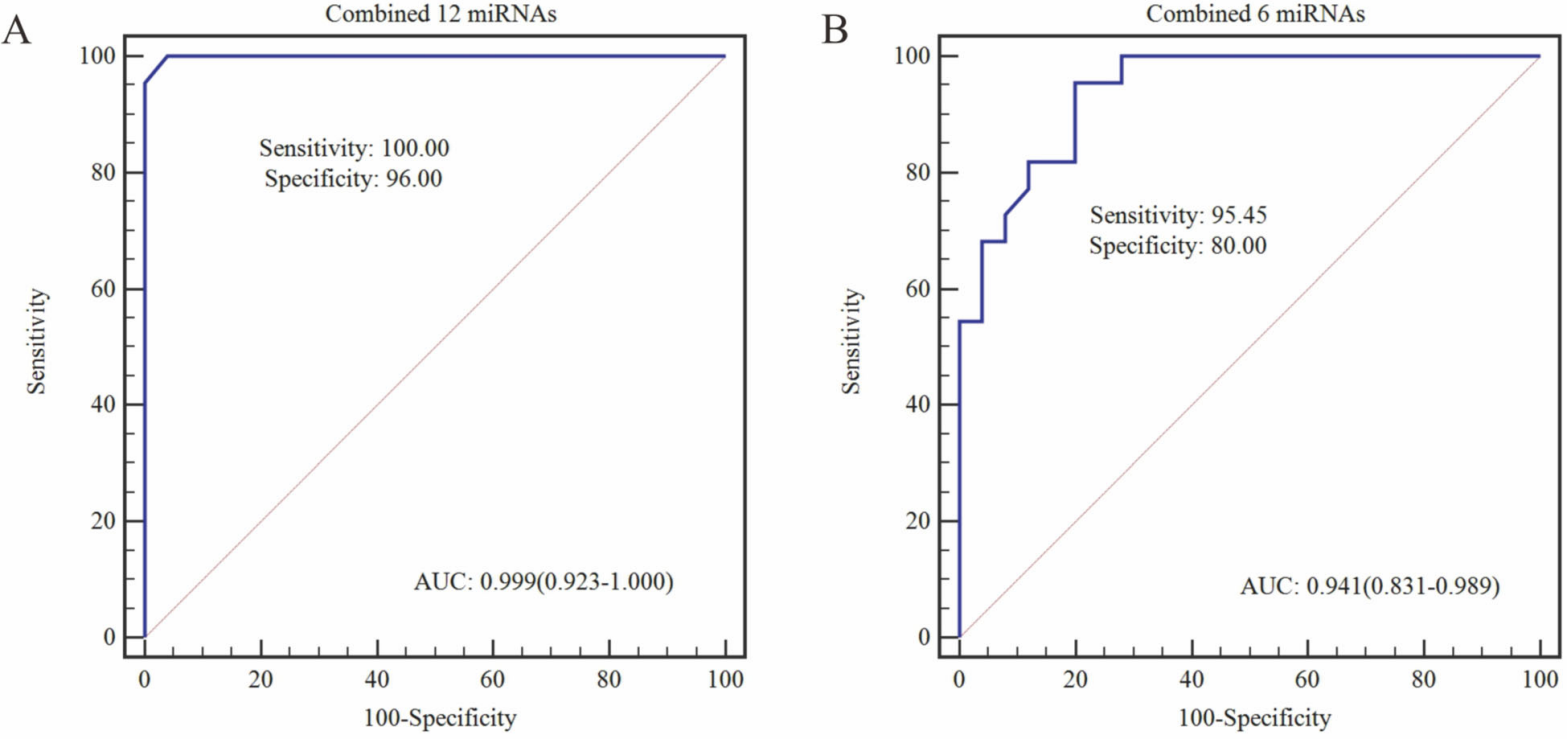
ROC curve of combined miRNA analysis. **a** ROC curve of 12 miRNAs (miR-30b-3p, miR-575, miR-650, miR-937-5p, miR-1202, miR-1203, miR-1321, miR-3612, miR-3714, miR-4259, miR-4478, and miR-4730) in discrimination between NPC patients and healthy controls, with an AUC of 0.999 and a sensitivity of 100.00% and a specificity of 96.00%. **b** A combined ROC curve of the 6 miRNAs (miR-30b-3p, miR-1202, miR-1321, miR-3612, miR-4478, and miR-4730) showed an AUC of 0.941, with a sensitivity of 95.45% and a specificity of 80.00%

The selected 12 saliva miRNAs were assessed by both ROC curve analyses and linear regression to determine the diagnostic power for NPC detection. The determined areas under the receiver operating characteristic curve (AUC) values of these 12 miRNAs for NPC diagnosis ranged from 0.764 (95% CI, 0.617–0.875) to 0.883 (95% CI, 0.755–0.958), respectively (Additional file 1: Figure S2). The results indicate that these 12 miRNAs have potential utility for diagnosis of NPC since all AUCs are > 0.75. The combined 12 miRNAs provides the best diagnostic accuracy in discrimination of NPC patients from healthy controls with an excellent AUC of 0.999 (95% CI, 0.923–1.000), an optimal sensitivity of 100.00%, as well as a specificity of 96.00% (Fig. 6a). A scoring approach employing the 6 significantly altered miRNAs (miR-30b-3p, miR-1202, miR-1321, miR-3612, miR-4478, and miR-4730) also revealed a good but lower diagnostic accuracy when compared to the 12 miRNAs score, with an AUC of 0.941 (95% CI, 0.718–0.938), sensitivity of 95.45% and specificity of 80.00% (Fig. 6b).

We categorized the miRNAs with diagnosis potential for NPC into three subgroups based on their expression patterns between various clinical stages (Additional file 1: Figure S3). In subgroup 1 (miR-937-5p, miR-4259, miR-1321, and miR-575), the four miRNA expressions dramatically decreased in stage I. In groups 2 (miR-3612, miR-30b-3p, miR-1202, miR-1203, and miR-4730) and 3 (miR-3714, miR-650, and miR-4478), miRNA expressions gradually decreased from normal to stage II: the differences between group 2 and 3 is that miRNA expressions slightly increased from stage II to stage IV in group 3. These results suggested that these miRNAs might play various roles in the stepwise development of NPC.

## Discussion

There is a growing body of evidence indicating that circulating miRNAs in the serum and plasma of NPC patients are potential non-invasive biomarkers [4, 21, 22]. However, data regarding miRNAs in saliva, as an extracellular fluid component, are not available for NPC patients. To our knowledge, this study is a first attempt to explore the feasibility of detecting NPC related miRNA levels in saliva and using specific saliva miRNA patterns as potential biomarkers for NPC. From the saliva specimens, we screened a panel of 12 miRNAs that exhibited remarkable alteration of expression level between the NPC patients and healthy controls. Furthermore, ROC analyses demonstrated an improvement of the diagnostic accuracy when the 12 miRNAs were interrogated together. For this miRNA panel, we were able to reach a discriminatory power of AUC = 0.999. When scoring with the 6 most altered miRNAs, the accuracy was high with an AUC of 0.941. This pilot study was designed as an initial step toward developing clinically applicable diagnostic biomarkers. The results of our study support further investigation in expanded cohorts.

Different technologies such as quantitative real-time PCR (qPCR) or microarray have been widely used for miRNA expression profiling. Moreover, data normalization using reference genes or optimal calculation methods for data correction is critical for quantification of miRNA transcripts. In qPCR, the extensively used housekeeping gene of tissue-based miRNA analyses is snRNA U6, while miR-1228, miR-30b-5p, and miR-16 are already implemented as potential housekeepers in plasma, serum, and urine [23–26]. Although these transcripts display constant expression in single analysis, their expression levels may be affected by specific types of disease or different experimental conditions [24]. Alternatively, the normalization strategies could take into account the total miRNA expression in the samples. In microarray-based techniques, information about the whole miRNA content of the sample is available and can be used to serve normalization. For the one-color SHUT microarray used in this study, we employed quantile normalization, which was proved to be ideal for one-channel miRNA microarray analysis [10], to detect distinct NPC-dependent salivary miRNA profiles. By doing so, we avoided additional validation of reference genes for salivary miRNAs since a single reference gene correction is insufficient to obtain reliable miRNA data.

In this study, the PPI network analysis indicated that the most significant hub gene is TP53, which is targeted by miR-937-5p. Other genes targeted by miR-4478 (PPM1D, CYCS, CASP8, and APAF1), miR-1321 (SESN2, RRM2, and CCND3), miR-3714 (CCND1), and miR-3612 (CCNB1) were also significantly enriched in p53 signaling pathway. The p53 protein is a well-know tumor suppressor encoded by TP53 and normally functions to inhibit the growth of aberrant cells by inducing growth arrest, DNA repair or apoptosis of the aberrant cell. It has been reported the p53 is short-lived and maintained at low levels in healthy cells, but overexpressed in most malignant tumors to help prevent cancer, including NPC [27, 28]. Therefore, decreased expression of the above miRNAs may play important roles in NPC by promoting p53 signaling pathway to exert anticancer function.

Moreover, target genes of miR-30b-3p (FOLR1, EHD2, and PDGFRA), miR-937-5p (SMAD2 and HSPA1B), miR-4478 (PARD6B and HSPA2), miR-3612 (PDGFRA and NEDD4L), miR-650 (PDGFRA and NEDD4L) and miR-575 (VPS36 and TSG101) were significantly enriched in the top pathway, endocytosis (Fig. 3b). EBV has long been recognized as a causative agent of NPC, and it enters nasopharyngeal epithelial cells via endocytosis [29]. Accordingly, increased expression levels of the endocytosis-related genes regulated by corresponding miRNAs in NPC patients are probably caused by EBV infection. It could be speculated that these miRNAs might play important roles in EBV entry into NPC cells by regulating their target genes that participate in endocytosis.

Due to the nature of this study, there are considerable limitations to our findings at the current stage. First, since all NPC patients involved were diagnosed at the stage of illness, this study lacks early stage patient data. Therefore, the value of the reported biomarker panel to distinguish individuals with onset cancer from healthy controls is yet to be determined. Second, this study is limited by the cohort size: more specimens are required to further validate the proposed miRNA panels. In addition, only NPC specimens are tested in this study. However, to fully assess the panel’s specificity to NPC, other cancer types especially the surrounding cancers such as esophageal cancer and oral cancer, must also be examined. Even so, the outcomes of this study attest to the potential of salivary miRNA panel to improve cancer diagnosis. Consequently, the necessity of addressing the aforementioned limitations with a more thorough research at a larger scale is adequately justified.

## Conclusions

Saliva collection is more convenient, noninvasive and cheaper than other sample collection methods, specifically blood collection, and it shows great promise in disease screening. In this study, we identified 12 significantly altered and specifically regulated miRNAs in saliva of NPC patients compared to healthy controls using our miRNA microarray platform. These miRNAs might play important roles in the pathogenesis of NPC by regulating their target genes. With this pilot study, we evaluated for the first time the feasibility of saliva miRNAs as novel, non-invasive diagnostic biomarkers for the detection of NPC. We anticipate that an expansion of the presented study would further confirm and substantiate these discoveries.

## Additional file

Additional file 1: **Table S1.** The dysregulated (down-regulated) miRNAs in the NPC samples with the cutoff criteria of *P* < 0.01 and |fold change| > 2. **Table S2.** Putative target genes of the dysregulated miRNAs in the saliva samples of NPC patients. **Table S3.** The enriched Gene Ontology (GO) terms in molecular function (MF), biological process (BP) and cellular component (CC) categories for target genes of all the 12 differentially expressed miRNAs. FDR: false discovery rate. **Table S4.** The top ten enriched pathways for target genes of all the 12 differentially expressed miRNAs. **Table S5.** The target genes with degrees not less than five in the protein-protein interaction network. **Table S6.** Sequences of RT primer, and PCR primers used for quantitative real-time PCR (qRT-PCR). **Table S7.** Detail information of protein-protein interactions from the Search Tool for the Retrieval of interacting Genes database (STRING) online**. Figure S1.** Validation of the miRNA expression (miR-937-5p, miR-650, miR-3612, miR-4478, miR-4259, miR-3714, miR-4730, miR-1203, miR-30b-3p, miR-1321, miR-1202, and miR-575) by qRT-PCR in 22 patients and 25 healthy controls. **Figure S2.** ROC curves of the diagnostic potential of the 12 individual salivary miRNAs (has-miR-30b-3p, has-miR-575, has-miR-650, has-miR-937-5p, has-miR-1202, has-miR-1203, has-miR-1321, has-miR-3612, has-miR-3714, has-miR-4259, has-miR-4478, and has-miR-4730) in discrimination between NPC patients and healthy controls. The AUC values ranged from 0.764 to 0.883, respectively. **Figure S3.** Diagnostic miRNA expressions were classified into 3 different patterns based on various clinical stages. (DOCX 1214 kb)

## Data Availability

The data analyzed during the current study are available from the corresponding author on reasonable request.

## Abbreviations

ACTB: Actin beta
ALB: Albumin
AUC: Areas under the receiver operating characteristic curve
CASP8: Caspase 8
CCNB1: Cyclin B1
CCND1: Cyclin D1
CRP: C-reaction protein
CYCS: Cytochrome c somatic
DAVID: Database for Annotation, Visualization and Integrated Discovery
EMT: Epithelial-to-mesenchymal transition
FDR: False discovery rate
FFPE: Formalin fixed paraffin-embedded
GO: Gene ontology
HGB: Hemoglobin
IKBKG: Inhibitor of nuclear factor kappa B kinase subunit gamma
IMRT: Intensity-modulated radiotherapy
JUN: Jun proto-oncogene AP-1 transcription factor subunit
LDH: Lactate dehydrogenase
MAPK: Mitogen-activated protein kinase
miRNAs: MicroRNAs
mRNA: Messenger RNA
NME2: NME/NM23 nucleoside diphosphate kinase 2
NPC: Nasopharyngeal carcinoma
PDGFRA: Platelet—derived growth factor receptor alpha
PPI: Protein-protein interaction
PPM1D: Protein phosphatase, Mg^2+^/Mn^2+^ dependent 1D
Pre-EBV DNA: Pre-treatment plasma Epstein-Barr Virus DNA
qPCR: Quantitative real-time PCR
RAC1: Ras-related C3 botulinum toxin substrate 1
ROC: Receiver operating characteristic
SHUT: Stacking-Hybridized Universal Tag
STRING: Search Tool for the Retrieval of interacting Genes database
TOB2: Transducer of receptor tyrosine-protein kinase
UICC/AJCC: International Union against Cancer/American Joint Committee on Cancer
UMPS: Uridine monophosphate synthetase
UT: Universal Tag
WHO: World health organization
XIAP: X-linked inhibitor of apoptosis protein
