# Supplementary material for: Genome-wide study of salivary microRNAs as potential noninvasive biomarkers for detection of nasopharyngeal carcinoma
Source: BMC Cancer. 2019 Aug 28;19:843. doi: 10.1186/s12885-019-6037-y (PMC6712819; doi:10.1186/s12885-019-6037-y)
Supplement: Supplementary file 1 — Table S1. The dysregulated (down-regulated) miRNAs in the NPC samples with the cutoff criteria of P < 0.01 and |fold change| > 2. Table S2. Putative target genes of the dysregulated miRNAs in the saliva samples of NPC patients. Table S3. The enriched Gene Ontology (GO) terms in molecular function (MF), biological process (BP) and cellular component (CC) categories for target genes of all the 12 differentially expressed miRNAs. FDR: false discovery rate. Table S4. The top ten enriched pathways for target genes of all the 12 differentially expressed miRNAs. Table S5. The target genes with degrees not less than five in the protein-protein interaction network. Table S6. Sequences of RT primer, and PCR primers used for quantitative real-time PCR (qRT-PCR). Table S7. Detail information of protein-protein interactions from the Search Tool for the Retrieval of interacting Genes database (STRING) online. Figure S1. Validation of the miRNA expression (miR-937-5p, miR-650, miR-3612, miR-4478, miR-4259, miR-3714, miR-4730, miR-1203, miR-30b-3p, miR-1321, miR-1202, and miR-575) by qRT-PCR in 22 patients and 25 healthy controls. Figure S2. ROC curves of the diagnostic potential of the 12 individual salivary miRNAs (has-miR-30b-3p, has-miR-575, has-miR-650, has-miR-937-5p, has-miR-1202, has-miR-1203, has-miR-1321, has-miR-3612, has-miR-3714, has-miR-4259, has-miR-4478, and has-miR-4730) in discrimination between NPC patients and healthy controls. The AUC values ranged from 0.764 to 0.883, respectively. Figure S3. Diagnostic miRNA expressions were classified into 3 different patterns based on various clinical stages. (DOCX 1214 kb) [file 12885_2019_6037_MOESM1_ESM.docx]

**Genome-wide study of salivary microRNAs as potential noninvasive biomarkers for detection of** **nasopharyngeal carcinoma**

Lirong Wu^1†^, Kexiao Zheng^2†^, Cheng Yan^2^, Xuan Pan^3^, Yatian Liu^1^, Juying Liu^1^, Feijiang Wang^1^, Wenjie Guo^1^, Xia He^1*^, Jiong Li^2*^, Ye Shen^2*^

^1^Department of Radiation Oncology, Jiangsu Cancer Hospital & Jiangsu Institute of Cancer Research & The Affiliated Cancer Hospital of Nanjing Medical University, Nanjing 210009, China

^2^Nano-Bio-Chem Centre, Suzhou Institute of Nano-Tech and Nano-Bionics, Chinese Academy of Sciences, Suzhou, 215123, China.

^3^Department of Oncology, Jiangsu Cancer Hospital & Jiangsu Institute of Cancer Research & The Affiliated Cancer Hospital of Nanjing Medical University, Nanjing 210009, China

†Contributed equally

*Corresponding author

**Additional file 1:**

**Table S1.** The dysregulated (down-regulated) miRNAs in the NPC samples with the cutoff criteria of P < 0.01 and |fold change| > 2.

| **Name** | **Fold change** | **P value** |
| --- | --- | --- |
| hsa-miR-937-5p | 0.37 | 3.61E-4 |
| hsa-miR-650 | 0.43 | 3.72E-4 |
| hsa-miR-3612 | 0.45 | 3.52E-4 |
| hsa-miR-4478 | 0.46 | 7.16E-4 |
| hsa-miR-4259 | 0.44 | 9.71E-3 |
| hsa-miR-3714 | 0.41 | 8.21E-3 |
| hsa-miR-4730 | 0.49 | 4.29E-3 |
| hsa-miR-1203 | 0.48 | 4.66E-3 |
| hsa-miR-30b-3p | 0.47 | 8.00E-4 |
| hsa-miR-1321 | 0.48 | 2.78E-3 |
| hsa-miR-1202 | 0.47 | 9.72E-6 |
| hsa-miR-575 | 0.49 | 7.20E-4 |

**Table S2.** Putative target genes of the dysregulated miRNAs in the saliva samples of NPC patients.

| **Name** | **Target genes** |
| --- | --- |
| hsa-miR-937-5p | FZNF623, ZNF573, XPO7, UBXN2A, UBN2, TXLNA, TRIM37, TP53, TMX4, TMEM98, TG, SURF4, ST6GAL1, SPPL2A, SPG20, SMAD2, SLC6A4, SLC35E2B, SHMT2, SEPHS1, SCRG1, SCNN1G, RPS16, RNF165, PROSC, PLD5, OGT, OGFRL1, NUTM2E, NKX6-1, NECAB3, NDOR1, NAV1, MTDH, MSRB1, METRN, MACC1, LUZP1, LILRB2, JMJD1C, IVD, INSIG1, ICOSLG, HSPA4L, HSPA1B, HSBP1, GEMIN4, FOS, FLVCR1, FAM168A, ERP44, EIF4EBP1, EFCAB11, DCTN6, CYB561A3, CRAMP1L, COX6B1, CORO2A, CLIC6, CENPN, CEBPG, CDKAL1, CD244, CD1D, CAPZA2, C9orf41, C6orf132, C17orf105, BCAS4, ANTXR2, AGO2, ACTB |
| hsa-miR-3714 | ZWINT, ZNF674, ZNF609, ZNF582, ZNF507, ZFYVE26, ZFP69B, ZBTB7B, WNT7B, WDR81, VANGL1, USP14, UHMK1, UBXN2B, UBE3C, TROVE2, TOM1L2, TMEM41A, TMEM181, TMEM167A, TMEM106B, TIPIN, TBC1D1, TAGLN, TAF13, STARD3, SSR1, SRPRB, SRP9, SRM, SPRTN, SNX18, SNRPD3, SNAP23, SMTNL2, SLC35E2, SLC30A7, SLC29A1, SLC16A1, SFT2D3, SERTAD3, SDE2, SDCBP, SCAMP4, SATB2, S1PR2, RNF41, RNF24, RNF157, RECK, RARA, RAB5B, QKI, PTPRF, PTBP3, PTBP1, PRSS45, PRR14L, POC1B-GALNT4, PLLP, PLIN3, PI4K2B, PHF15, PCCB, PAX5, PATL1, PARP16, PANK2, OTUD1, NFIX, NFIB, NEK4, NDUFA7, NCR3LG1, NCMAP, MYLIP, MTFMT, MTDH, MRPL49, MARVELD2, MAN2B2, MALT1, LYSMD3, LRRC1, LPCAT3, LAX1, LASP1, KLHL42, KLHL28, KLHL15, KIAA1328, ING1, IL17RB, IFNGR1, IFNAR2, ICMT HSPA6, HSD17B12, HIATL1, HEPHL1, HECTD3, GXYLT2, GXYLT1, GTF2E1, GOSR1, GNAT1, GLO1, GJD2, GGCX, GALNT4, G3BP1, FZR1, FSTL3, FBXO28, FAM91A1, FAM186A, FAM131B, EVI5, ERCC6L2, EPM2AIP1, EFNA1, EFCAB14, DYRK3, DPYSL5, DNAJC10, DLGAP3, DDX52, DDI2, CYSLTR2, CTNND1, CRTAP, CREBRF, CPEB3, COMMD5, COL13A1, CHSY1, CFL2, CES2, CD1D, CCND1, CCDC137, C9orf41, C5orf45, C5ORF24, C20orf27, C1QTNF6, BZW1, BTRC, BTG2, BAHD1, B4GALNT3, ATXN3, ASB16, ARL5B, ARHGAP1, ANP32E, ANKRD53, ANKRD11, AMOTL2, AMOTL1, AK4, ADRB3 |
| hsa-miR-650 | ZSCAN29, ZNRF3, ZNF716, ZNF70, ZNF69, ZNF527, ZNF490, ZNF317, ZNF264, ZNF117, ZFP62, ZER1, USH1G, UGGT1, TRIOBP, TRIM67, TRIM65, TRAPPC2, TMOD2, TMEM214, TMEM209, TMEM184C, THBS1, TBL2, TAF8, SVOP, STK17B, SPC24, SNAP23, SMG1, SMARCD1, SLC29A1, SLC27A1, SIGLEC14, SERBP1, SENP5, SCARF1, SCAMP4, SAMD5, RSL1D1, RPL28, RDH13, RASSF2, RAC1, PTDSS2, PSD4, PNO1, PNMAL2, PLCXD1, PIGR, PFAS, PEG10, PDGFRA, PDE11A, PCSK9, PCCB, PATZ1, OTUD5, NT5DC3, NFAT5, NEDD4L, NECAB3, NARF, MRVI1, MPHOSPH8, MORN4, LUC7L, LETMD1, LENG8, KLHL26, KATNAL1, JOSD1, ITGA11, IREB2, IQSEC2, IPCEF1, IMMP2L, HRH4, HOOK3, HMOX1, HIRIP3, HHIPL1, GUF1, GSR, GNS, GNG4, GLG1, GALNT2, GALNT11, FKBP5, FEM1A, FBLIM1, F2RL2, DUSP19, DNAL1, DHX40, DDX19A, CYP8B1, CYP51A1, CYP20A1, CSDC2, CRLF3, CRKL, CRISPLD2, CPNE5, CPA4, COX18, CLCN5, CIITA, CENPN, CDCP1, CARD8, BTG2, BMP8A, BCAS4, ATP6V0A2, ASB6, ANAPC16, ALDOA, ADRBK1, ADD2, ACTR1A, ACOX1 |
| hsa-miR-4259 | WFDC6, VAT1, UBBP4, UBB, UBALD1, TSPYL1, TNRC6A, TMED2, STK38, SSR3, SRCAP, SPRY4, SLC36A1, SLC10A7, SIX5, RCE1, POU2F1, PHAX, NDRG1, MSC, MRRF, MEN1, MAX, LIMA1, KCTD10, HSD11B1L, HNRNPUL1, H3F3B, GTF3C4, GPX1, FOLR1, FAM168A, FAM127B, DSN1, DR1, DNAL1, CTNND1, CBX4, B4GALT7, AP4S1 |
| hsa-miR-3612 | ZSWIM6, ZSCAN29, ZNRF3, ZNF749, ZNF716, ZNF70, ZNF69, ZNF669, ZNF581, ZNF573, ZNF527, ZNF490, ZNF451, ZNF383, ZNF317, ZNF264, ZNF117, ZFP62, ZFAND4, ZER1, ZC3H8, USH1G, UGGT1, UBR1, TTC39B, TRIOBP, TRIM72, TRIM67, TRIM65, TRAPPC2, TNFRSF13C, TMOD2, TMEM81, TMEM67, TMEM214, TMEM209, TMEM184C, TLCD2, TIPIN, TIMM8A, TIMM10B, TIFA, THEM6, THBS1, TG, TFDP2, TBL2, TAF8, SVOP, STK17B, SSBP2, SRRD, SPPL2A, SPC24, SNRPD1, SNAP23, SMS, SMG1, SMARCD1, SLFN12L, SLC35F6, SLC29A1, SLC27A1, SKIL, SIGLEC9, SIGLEC14, SGPP2, SERBP1, SENP5, SDR9C7, SCRG1, SCARF1, SCAMP4, SAMD5, RSL1D1, RPL28, REXO2, RDH13, RASSF2, RAC1, PTPLAD2, PTDSS2, PSD4, PRICKLE1, PPIL4, PNO1, PNMAL2, PLCXD1, PLA2G16, PIGR, PHAX, PFAS, PEG10, PDGFRA, PDGFRA, PDE11A PCSK9, PCCB, PATZ1, PARP2, PAPOLA, OTUD5, NT5DC3, NPY4R, NME2, NME1-NME2, NIF3L1, NFAT5, NEDD4L, NECAB3, NDUFA7, NARF, MTMR12, MTHFD2, MRVI1, MRPS14, MRPS10, MRPL12, MPLKIP, MPHOSPH8, MORN4, METTL14, MCUR1, MARS2, LYRM4, LUC7L, LIPC, LETMD1, LENG8, KLHL26, KLHL23, KIF6, KIAA1958, KIAA1551, KIAA1456, KIAA0391, KATNAL1, JOSD1, ITGA11, IREB2, IQSEC2, IPCEF1, IMMP2L, IFNLR1, IBA57, HSPA6, HRH4, HOOK3, HMOX1, HIRIP3, HINT1, HHIPL1, GUF1, GSR, GPR156, GNS, GNG4, GLUL, GLG1, GLCCI1, GIGYF1, GEMIN6, GDE1, GALNT2, GALNT11, GABRR2, FKBP5, FEM1A, FBLIM1, FAM83B, FADS6, F2RL2, ESF1, EPGN, EIF4EBP1, EFNB1, DUSP19, DNAL1, DHX40, DEFB105B, DEFB105A, DDX19A, DCPS, CYP8B1, CYP51A1, CYP20A1, CTSL2, CSDC2, CRLF3, CRKL, CRISPLD2, CPNE5, CPE, CPA4, COX18, CLSPN, CLPSL1, CLCN5, CKAP2L, CIITA, CENPN, CDCP1, CDC5L, CCNB1, CCDC80, CCBE1, CARD8, C7orf73, C17orf85, C10orf71, BTG2, BPTF, BMP8A, BCAS4, B4GALT7, ATP6V0A2, ATP5S, ASB6, ARSK, AR AP5M1, ANAPC16, ALDOA, ADRBK1, ADD2, ACTR1A, ACSBG1, ACOX1 |
| hsa-miR-4478 | ZSWIM1, ZSCAN16, ZNF99, ZNF891, ZNF805, ZNF783, ZNF770, ZNF766, ZNF749, ZNF738, ZNF701, ZNF695, ZNF681, ZNF669, ZNF665, ZNF641, ZNF639, ZNF638, ZNF623, ZNF619, ZNF589, ZNF587, ZNF585B, ZNF584, ZNF583, ZNF581, ZNF566, ZNF556, ZNF555, ZNF500, ZNF439, ZNF43, ZNF417, ZNF33A, ZNF141, ZNF124, ZMYM1, ZDHHC8, ZBTB8OS, ZBTB8B, ZBED3, YIPF4, YES1, WSB1, WIZ, WEE1, VAMP1, UTP15, URM1, UQCRQ, UNC5C, UMPS, ULBP3, UGGT1, UBXN2A, TXNRD2, TXNIP, TXNDC16, TVP23C, TSPYL1, TSPAN15, TRUB2, TRPM7, TRIM65, TRIM56, TRAPPC2, TRAF1, TPM3, TNPO3, TNFSF15, TMTC1, TMEM59, TMEM216, TMEM185B, TMEM145, TMEM145, TMED9, TMCO1, TMBIM6, TLE3, TIAL1, THAP1, TES, TERF2, TCF23, TBXA2R, TBC1D19, SYT15, SYNJ2BP, SUMF2, STX7, STK17B, STAT2, SSBP2, SRD5A1, SPAST, SOAT1, SNX27, SNRPD1, SMAD9, SLX4IP, SLPI, SLFN12L, SLC7A5P2, SLC39A9, SLC38A7, SLC36A2, SLC35F1, SLC35E3, SLC35D2, SLC30A6, SLC2A5, SLC29A4, SLC27A1, SLC25A45, SLC19A3, SLC16A4, SLC15A1, SLC11A2, SLC10A6, SIKE1, SHOX, SHOC2, SHISA9, SEPHS1, SCNM1, SCARF1, SAR1A, RSRC1, RPP14, RPH3AL, RPF2, RNASEH2B, RGS9BP, RFT1, RFC2, RABAC1, RAB4A, RAB36, RAB13, QDPR, PURB, PTRH2, PTPLB, PTBP2, PSMC4, PSD4, PRIM2, PRIM1, PRICKLE4, PPY, PPP1R15B, PPM1L, PPM1D, PPEF2, POLR3K, PMPCA, PLEKHG2, PLEKHB2, PLEKHA1, PLD5, PLCE1, PLA2G4A, PHACTR4, PGPEP1, PGAM5, PEX26, PEX13, PER2, PDPN, PDP2, PDE12, PCGF3, PCDHA6, PAX6, PARVB, PARP2, PARK7, PARD6B, PAQR7, PAK1IP1, PADI1, PACS2, OTUD7B, OLFML2A, OGFRL1, NXN, NUP85, NTPCR, NRXN3, NR2F6, NPR1, NOTO, NMUR1, NLRC3, NFYA, NEK9, NDUFS5, NDRG3, NAA50, N6AMT1, MYH9, MXRA7, MUC20, MTMR10, MSL2, MRPS30, MRPS16, MRPL49, MRPL12, MRI1, MRE11A, MPPE1 MOB3A, MINOS1, MICA, MGAM, MFSD4, MEMO1, MED17, MCF2L2, MBL2, MAPKAPK5, MAPK1IP1L, MANSC1, MAK, LY6G5B, LRRC47, LRP10, LLGL1, LIX1L, LIPC, LINC00598, LINC00346, LIN7C, LHPP, KRT8, KLLN, KLHL23, KLHL21, KIF6, KIAA1328, ITGA3, IRGQ, IREB2, IRAK4, IPCEF1, INADL, ILDR1, IL17REL, IKZF3, IKBKG, IGF1, HUS1, HSPA2, HSP90B1, HOXB3, HOOK3, HJURP, HEYL, GXYLT2, GTPBP10, GSTCD, GSG2, GRSF1, GPR75, GOLGA3, GLUL, GLCE, GINS2, GEMIN4, GDE1, GABPB1, FUT2, FOSL2, FKTN, FBXW8, FBXW2, FBXO45, FAM60A, FAM208A, FAM118A, EXOSC6, EXOSC2, EXOC8, EVI5, EVC, ERN1, ENTPD5, ENAH, EMILIN2, ELMSAN1, EIF4A3, EIF2S3, EIF2AK2, EFCAB11, DTD2, DSCR3, DNAJC28, DNAJC10, DNAH9, DNA2, DHODH, DFFB, DFFA, DCTN6, DARS2, CYCS, CXorf56 CSTF1, CRKL, CRISPLD2, CRCP, CPE, COX6B1, CLPB, CLOCK, CLCN7, CLCC1, CKS1B, CHORDC1, CHMP1B, CERS2, CEP57L1, CENPN, CENPM, CDKAL1, CDK19, CDCP1, CDC73, CD3EAP, CD300LG, CD209, CD1D, CCL22, CCDC77, CBFA2T2, CASP8, CASP16, CARD6, CAMLG, CACNG8, C9orf156, C6orf141, C5orf45, C5AR2, C3, C2orf48, C1orf210, C1orf174, C14orf142, C11orf84, C10orf111, BTD, BTBD19, BROX, BRI3BP, BPNT1, BLOC1S3, BCL9L, BAZ2A, ATP13A4, ATP11C, ARSK, ARSA, ARL8B, ARHGAP11A, AQP6, APTX, APOL6, APAF1, AP1S3, ANP32E, ANKRD40, ANGPT4, ALG10B, ALDOA, AKNA, AKAP6, AK4, AGXT2, AGBL5, AGAP9, ACOT2, ACBD7, ABHD17B, ABHD15, ABCF1, AAED1 |
| hsa-miR-30b-3p | ZYG11A, ZSCAN29, ZNF878, ZNF860, ZNF770, ZNF749, ZNF708, ZNF703, ZNF701, ZNF70, ZNF695, ZNF681, ZNF674, ZNF665, ZNF641, ZNF607, ZNF584, ZNF573, ZNF557, ZNF548, ZNF503, ZNF500, ZNF491, ZNF490, ZNF460, ZNF451, ZNF417, ZNF394, ZNF324B, ZNF317, ZNF284, ZNF280C, ZNF264, ZNF250, ZNF17, ZNF124, ZNF117, ZMYM1, ZKSCAN3, ZFP69B, ZFAND4, ZCCHC8, ZCCHC24, ZBTB37, YME1L1, YAE1D1, XRCC6, XKR4, XIAP, WWTR1, WSB1, WNT7B, WIZ, WHAMM, WDR75, WDR55, WARS, UST, UQCRQ, UPK3BL, ULBP3, UGGT1, UBXN2B, UBXN2A, UBLCP1, UBE2D4, UBE2B, TTLL9, TTLL1, TTC9C, TTC39B, TTC21B, TSPYL4, TRUB2, TRIM66, TRIM65, TRIM13, TRIB1, TRAF3IP2, TPCN2, TOX4, TOR1AIP2, TOMM20, TNS4, TNRC6A, TNFRSF13C, TMTC1, TMPRSS12, TMPRSS11BNL, TMEM91, TMEM63C, TMEM41B, TMEM33, TMEM251, TMEM239, TMEM209, TMEM184C, TMEM167A, TMEM167A, TMEM120B, TMEM11, TM6SF2, TLN1, TLCD2, TJAP1, TIMM50, TIAL1, THAP5, TFIP11, TES, TDGF1P3, TBXA2R, TBC1D19, TANGO2, TACO1, SYTL3, SYNJ2, SUSD1, SUMF2, SUGT1, STRIP2, STK4, STK38, SSR3, SSR1, SSBP2, SRRD, SPTLC2, SPATA5, SPAST, SNX27, SNX1, SNTN, SNRPD3, SNRPD1, SMS, SMAD9, SLFN12L, SLC7A5, SLC38A7, SLC35F6, SLC35E2, SLC29A4, SLC27A1, SLC25A45, SLC25A34, SLC1A5, SLC1A2, SLC19A3, SLC16A4, SLC11A2, SLC10A6, SIT1, SIPA1, SIGLEC14, SGTB, SFT2D2, SF3B3, SERAC1, SCUBE3, SCNM1, SCD5, SCAND3, SCAMP4, SAR1A, RRP7A, RRAD, RPRD2, RPP30, RPL7L1, RPH3AL, RORC, RNPS1, RNF24, RMDN1, RHOF, RFTN2, RFT1, RBMS2, RBM8A, RBM48, RBBP4, RASSF9, RARA, RAP2B, RAD51B, RAD51, RAB4A, RAB36, RAB33B, RAB2B, QPCTL, QDPR, PURB, PTRH2, PRRG4, PRKAR2A, PRIM2, PRICKLE1, PPTC7, PPM1L, PPM1D, PPEF2, POLR3K, POLR3D, POLR2J3, PNPLA6, PNMA2, PMPCA, PLXND1, PLLP, PLEKHH1, PLEKHB2, PLEKHA1, PLD6, PLCE1, PLAGL2, PITPNA, PIM3, PIGR, PHF12, PHC1, PHAX, PHACTR4, PGPEP1, PGM2L1, PGAM5, PGAM4, PFKFB3, PDGFRA, PDE7B, PDE7A, PCNP, PCDHB11, PAQR5, PAPOLA, OSBPL2, OSBPL10, OPA3, ONECUT3, NUP43, NUP155, NUDT7, NUDT19, NRF1, NPR1, NOA1, NKRF, NKAP, NHLRC2, NDUFS5, NDUFA7, NDUFA4P1, NCOR2, NCMAP, NCKIPSD, NAV1, NANOS1, MYH9, MYH11, MYBBP1A, MYADM, MTMR10, MTL5, MSRB2, MSN, MSL2, MRPS16, MRPL12, MRO, MPLKIP, MORN4, MOCS3, MOB1B, MMAB, MLLT1, MINOS1, MIER3, MFSD4, METTL14, MED28, MED16, MCF2L2, MBOAT2, MBL2, MBD6, MAZ, MAT2A, MARCH6, MANSC1, LY6G5B, LSG1, LRRC47, LRRC20, LRIG2, LONRF2, LLGL1, LINC00598, LINC00346, LAX1, KRT8, KLLN, KLHL26, KLHL21, KLHDC8A, KLHDC3, KLF8, KIF1C, KIAA1456, KIAA1328, KIAA1210, KIAA0754, KIAA0754, KHSRP, KBTBD12, KAT7, KANSL1, ITGB3, ITGA3, IRAK3, IQSEC3, INTS7, INMT, ING1, IKZF3 IGSF6, IGF1, IBA57, HSD17B12, HOXC4, HOOK3, HNRNPUL1, HIST1H2BD, HINT1, HES6, HECTD3, HAVCR2, HAUS3, GPRC5C, GPKOW, GPC4, GPBP1, GP5, GOLGA3, GNG4, GLUL, GLP2R, GIGYF1, GHITM, GGCX, GEMIN4, GCNT4, GCFC2, GCDH, GAPVD1, FYTTD1, FUT2, FTO, FRRS1, FOSL2, FOPNL, FOLR1, FNIP1, FNDC3B, FGFR1OP, FEM1A, FBXW8, FBXW2, FBXO45, FBXL18, FAT3, FAM83B, FAM73B, FAM71F2, FAM208A, FAM105A, FADS6, F2RL2, EXTL3, EXOSC2, EXOC8, EVI5, ETV3, ESCO2, ERCC6L2, EPHB2, EPG5, EIF4A3, EHD2, EFNB1, EBNA1BP2, DYRK2, DUSP28, DUSP19, DTD2, DSN1, DSEL, DPYSL5, DPM2, DPH2, DOCK7, DNAL1, DNAJC8, DNAJC24, DNAJC10, DNAH17, DNAH10OS, DISC1, DHX40, DHODH, DHDDS, DFNB59, DFFA, DESI1, DDX19A, CYP2W1, CYP20A1, CXXC4, CTNND1, CTDNEP1, CSTF1, CRTAP, CRLF3, CRKL, CRISPLD2, CRIPT, CRCP, CPSF2, CPM, COX6B1, COX18, COL5A1, COL4A3BP, CLSTN1, CLSPN, CLEC7A, CLCC1, CIAPIN1, CHORDC1, CHMP1B, CES3, CEP97, CEP104, CECR1, CDKAL1, CDH7, CD3D, CCNF, CCL22, CCDC77, CBY3, CAPZB, CAPN7, CAMLG, CACNG8, C5orf45, C3, C19orf47, C11orf58, C10orf76, BVES, BTNL3, BTF3L4, BRI3BP, BPNT1, BMP3, BEST3, BAK1, B3GALT5, ATP6V1A, ATP6AP1, ATP1B4, ATL3, ATF6, ASXL2, ARSK, ARPC3, ARL8B, ARL5B, ARL10, AQR, AQP6, APOBEC3F, ANKRD40, ANKFY1, ANGPT4, ALG10B, AKIP1, AHR, AGXT2, AGBL5, AGAP9, ADIPOQ, ADAR, ADAMTS4, ACAP2, ACADL, ACAA2, ABI2, AAED1 |
| hsa-miR-1203 | TONSL, TNFRSF13C, TMEM127, SUMO2, SMG7, SLC12A7, SAMD15, RTN4RL2, RRP7A, PIAS4, PDE4C, NUBP1, NPAS1, MSC, KPNA2, INTS7, ICA1L, EIF2B5, DLL4, CRISPLD2, CNNM4, CDH7, C10orf111, ATXN1, ARL5C, MGAM |
| hsa-miR-1202 | ZWINT, ZNF786, ZNF28, ZDHHC18, ZBTB43, WNT9A, WDR41, TXNIP, TNFRSF10D, TMEM2, TMEM184B, TMC5, TET3, SYNJ2BP, SVOP, SLC16A1, SIT1, SFMBT2, SDR16C5, RPUSD2, RNF11, RBM28, RBBP4, PRPF40A, PPIB, PAK7, NOL4, NGDN, MRPS23, MEAF6, MARCH9, LDLRAD4, KLHL15, KIAA1958, KBTBD11, GRM4, ETS1, ERVMER34-1, DPP8, DNAJA1, CTNND1, CSDE1, CHAC1, CBS, C9orf170, C20orf103, BTBD1, BAZ2A, BAG4, ARPC2, APOL6, ADD3 , ACTG1 |
| hsa-miR-1321 | ZNF710, ZNF70, ZNF566, ZNF34, ZNF134, ZFP36L1, ZFHX3, ZCCHC3, ZC3HAV1L, ZBTB34, WNT3, VCL, UPK3BL, UCP2, UBL4A, UBE2S, TUBB2A, TTF2, TSPAN11, TRIM67, TRIM44, TPM3, TOB2, TNRC6B, TMPRSS5, TMEM184A, TMEM138, TJP3, THRA, THBS1, TBC1D25, TAGLN, SUMO1, SUGT1, STK35, STARD3, ST7L, SRP19, SRCIN1, SP2, SORCS2, SNAP23, SLC30A7, SLC2A4, SLC29A4, SLC29A2, SLC29A1, SLC27A1, SLC26A9, SIX3, SH3TC2, SH3RF1, SESN2, SEPT2, SDC4, SCO1, SCD, SAMD10, RTKN, RRM2, RPRD2, RPH3A, RNF157, RNF111, RGS6, REXO2, RARA, RAB7A, PTDSS2, POLR2J3, POLR2F, POFUT1, PNPLA6, PITPNM3, PHB2, PGAM4, PEX11B, PDPR, PCYT1A, PAX5, PAX2, PARP11, OTUD7B, OLFML2A, NUFIP2, NTN1, NRG4, NRF1, NOL10, NKRF, NGFR, NFIX, NFAT5, NEK4, NCOR2, NAV2, NACC1, MYH14, MXRA7, MORC4, MLXIP, MLLT1, MINK1, MIDN, MAT2A, MAG, LY6H, LONRF3, LETMD1, KRT80, KLF13, KIF2C, KHSRP, KCNA5, JUN, ITGA11, IQSEC2, IPCEF1, IP6K1, IKBKG, IGFBP4, IFNAR2, ICOSLG, HOXB5, HOXB13, HMGXB4, HHIP, HES6, HDLBP, HACE1, GTF3C3, GPR107, GNG3, GNB1L, GLYR1, GIGYF1, GCDH, GATA6, FKBP5, FAM210A, FAM198A, FAAH, ENTPD7, DUSP9, DUSP7, DUSP28, DTHD1, DESI1, DDN, DCAF7, CYLD, CTDSP2, CRTC2, CNOT6L, CNBP, CLSTN1, CLASP1, CIITA, CHRDL1, CES2, CENPQ, CDKAL1, CDK14, CCND3, CCL11, CCDC108, CAPN15, CALR, C20orf96, C1QTNF6, C19orf47, C11orf84, C10orf55, BTG2, BRIX1, BCL2L13, BARHL1, ATXN7L3, ATP6V1B1, ATP6V1A, ATP2A3, ASXL1, ASB16, ARSA, ARL4C, ARHGAP31, APH1A, ANKRD45, ANKFY1, ALDH9A1, AKR7A2, AKAP11, AK2, AIP, AGXT2, AGO2, AEN, ACVR1B, ACTR2 |
| hsa-miR-4730 | ZNF33A, ZNF264, SLC47A1, RPS28, RANGAP1, RAB40C, PTPRF, MRI1, MICAL2, LY6H, LACE1, GPRC5C, GPR37L1, FAM228A, ARHGAP31 |
| hsa-miR-575 | ZRANB3, ZNF680, ZNF677, ZNF556, ZNF516, ZNF329, ZNF212, ZC3H12A, XPNPEP3, XIAP, WIPI2, WDR92, VPS36, USP1, UEVLD, UBXN2A, UBN2, TTC22, TSG101, TRAF3IP2, SZRD1, SYNPO2L, SOWAHC, SNRPD3, SNRNP48, SNAPIN, SMU1, SMIM12, SMG1, SLC43A1, SLC25A32, SIK2, SHROOM4, SHOC2, RDH10, RBM23, RBFOX2, RAD51, QPCTL, PTPRF, PTCD2, PRKX, PPP1R3B, POLR2F, POLD3, PLCG2, PITX3, OTUD5, ORAI2, NOLC1, NCAPG, MYOZ2, MYO10, MXD1, MSRB3, MRPS23, MKLN1, MEF2D, MED28, MEAF6, MAVS, LSG1, LIX1L, KREMEN1, KLF6, KIR3DX1, KIF1C, KIAA1467, KCNIP2, ITPRIPL2, IFNLR1, ICA1L, HIAT1, GRWD1, FAM118A, DYNAP, DUSP2, DHX33, DGKH, DCTN3, DCAF16, CRCP, COX15, CNNM4, CNKSR3, CMTM4, CLPP, CHST6, CHCHD4, CDC45, CD300LG, CCS, CCNA2, CCDC69, C20orf144, C19orf52, C17orf85, BTG2, BRMS1L, BLOC1S3, BCL2L1, BBX, ATP2A2, ATAD5, API5, ALG14, AKNA, ADRBK1, ABHD14B |

**Table S3.** The enriched Gene Ontology (GO) terms in molecular function (MF), biological process (BP) and cellular component (CC) categories for target genes of all the 12 differentially expressed miRNAs. FDR: false discovery rate.

| **GO_ID** | **GO_term** | **Category** | **Count** | **Target Genes** | **FDR** |
| --- | --- | --- | --- | --- | --- |
| GO:0006355 | regulation of transcription, DNA-templated | BP | 150 | VPS36, TP53, NME2, NME1-NME2, MAX, HNRNPUL1, CIITA, CALR, ATXN3 | 1.35E-2 |
| GO:0006351 | transcription, DNA-templated | BP | 186 | VPS36, TP53, STAT2, SMAD2, POLR2J3, POLR2F, NME2, NME1-NME2, IKBKG, HNRNPUL1, EIF2AK2, CIITA, CCND1, ATXN3, ATF6 | 1.71E-2 |
| GO:0005634 | nucleus | CC | 455 | XIAP, VPS36, UMPS, TUBB2A, TP53, STAT2, SMAD2, SESN2, RRM2, RAC1, PPM1D, POLR2J3, POLR2F, POLD3, PDGFRA, PARD6B, NME2, NME1-NME2, NEDD4L, MSN, MAX, KPNA2, JUN, ITGB3, IRAK4, IKBKG, HSPA4L, HSPA2, HSP90B1, HNRNPUL1, FOLR1, EIF2AK2, EHD2, DNAJA1, DFFB, DFFA, CYCS, CCND3, CCND1, CCNB1, CALR, ATXN3, ATF6, APAF1, ADRB3, ADAR, ACTG1 | 1.09E-4 |
| GO:0005654 | nucleoplasm | CC | 247 | XIAP, TP53, STAT2, SMAD2, RRM2, PRIM2, PRIM1, POLR3K, POLR3D, POLR2F, POLD3, NEDD4L, MAX, KPNA2, JUN, HSPA1B, HNRNPUL1, DHODH, DFFB, DFFA, CLEC7A, CKS1B, CIITA, CCND3, CCND1, CCNB1, CASP8, ATXN3, ATF6, ADAR, ACTB | 1.01E-2 |
| GO:0005730 | nucleolus | CC | 92 | TSG101, TP53, POLR3K, POLR2F, DFFB, ADAR | 2.48E-2 |
| GO:0003676 | nucleic acid binding | MF | 108 | POLR3K, EHD2 | 5.03E-3 |
| GO:0003677 | DNA binding | MF | 161 | TSG101, TP53, STAT2, SMAD2, PRIM2, POLR3D, POLR2J3, POLR2F, NME2, NME1-NME2, JUN, DFFB, CIITA, CALR, ADAR | 3.80E-2 |

**Table S4.** The top ten enriched pathways for target genes of all the 12 differentially expressed miRNAs.

| **Pathway_ID** | **Name** | **Count** | **Target Genes** | **FDR** |
| --- | --- | --- | --- | --- |
| hsa04144 | Endocytosis | 27 | VPS36, TSG101, SMAD2, PDGFRA, PARD6B, NEDD4L, HSPA2, HSPA1B, FOLR1, EHD2, ADRB3 | 1.07E-9 |
| hsa00230 | Purine metabolism | 21 | RRM2, PRIM2, PRIM1, POLR3K, POLR3D, POLR2J3, POLR2F, POLD3, NME2, NME1-NME2 | 7.01E-8 |
| hsa05162 | Measles | 17 | TP53, STAT2, MSN, IRAK4, HSPA2, HSPA1B, EIF2AK2, CCND3, CCND1, ADAR | 2.95E-6 |
| hsa04145 | Phagosome | 18 | TUBB2A, RAC1, ITGB3, CLEC7A, CALR, ACTG1, ACTB | 3.12E-6 |
| hsa05164 | Influenza A | 18 | STAT2, KPNA2, JUN, IRAK4, HSPA2, HSPA1B, HNRNPUL1, EIF2AK2, CYCS, CIITA, ADAR, ACTG1, ACTB | 2.24E-5 |
| hsa04115 | p53 signaling pathway | 12 | TP53, SESN2, RRM2, PPM1D, CYCS, CCND3, CCND1, CCNB1, CASP8, APAF1 | 4.03E-5 |
| hsa00240 | Pyrimidine metabolism | 14 | UMPS, RRM2, PRIM2, PRIM1, POLR3K, POLR3D, POLR2J3, POLR2F, POLD3, NME2, NME1-NME2, DHODH | 6.42E-5 |
| hsa04141 | Protein processing in endoplasmic reticulum | 17 | HSPA4L, HSPA2, HSPA1B, HSP90B1, EIF2AK2, DNAJA1, CALR, ATXN3, ATF6 | 9.51E-5 |
| hsa04210 | Apoptosis | 10 | XIAP, TP53, IKBKG, DFFB, DFFA, CYCS, CASP8, APAF1 | 2.23E-3 |
| hsa05222 | Small cell lung cancer | 11 | XIAP, TP53, MAX, IKBKG, CYCS, CKS1B, CCND1, APAF1 | 4.22E-3 |

**Table S5.** The target genes with degrees not less than five in the protein-protein interaction network.

| **Target genes** | **Degree** |
| --- | --- |
| TP53 | 28 |
| JUN | 17 |
| UMPS | 15 |
| CCND1 | 15 |
| ACTB | 14 |
| CASP8 | 14 |
| CCNB1 | 14 |
| CYCS | 14 |
| NME2 | 13 |
| POLR2F | 11 |
| APAF1 | 10 |
| NME1-NME2 | 10 |
| HSP90B1 | 10 |
| XIAP | 9 |
| PRIM1 | 8 |
| CKS1B | 7 |
| IKBKG | 7 |
| ATF6 | 7 |
| RAC1 | 7 |
| RRM2 | 7 |
| DNAJA1 | 7 |
| HSPA1B | 7 |
| ACTG1 | 7 |
| POLR3D | 6 |
| HSPA4L | 6 |
| KPNA2 | 6 |
| CCND3 | 6 |
| POLR3K | 6 |
| PDGFRA | 6 |
| CALR | 5 |
| MAX | 5 |
| MSN | 5 |
| POLR2J3 | 5 |
| DFFA | 5 |
| SMAD2 | 5 |
| EIF2AK2 | 5 |

**Table S6.** Sequences of RT primer, and PCR primers used for quantitative real-time PCR (qRT-PCR).

| **Name** | **Sequence (5'-3')** |
| --- | --- |
| RT primer | GCGAGCACAGAATTAATACGACTCACTATAGG(T)_12_VN |
| Universal reverse primer | GCGAGCACAGAATTAATACGAC |
| miR-937-5p-F | GTGAGTCAGGGTGGGG |
| miR-650-F | GGCAGCGCTCTCAGGAC |
| miR-3612-F | AGGCATCTTGAGAAAT |
| miR-4478-F | GAGGCTGAGCTGAGGAG |
| miR-4259-F | GTTGGGTCTAGGGGTCAGG |
| miR-3714-F | AAGGCAGCAGTGCTCC |
| miR-1203-F | CGGAGCCAGGATGCAG |
| miR-30b-3p-F | GGAGGTGGATGTTTACTT |
| miR-1321-F | CAGGGAGGTGAATGTG |
| miR-1202-F | AGCTGCAGTGGGGGAG |
| miR-4730 | CTGGCGGAGCCCATTCCA |
| miR-575-F | GAGCCAGTTGGACAGGAG |
| cel-miR-39-3p | CGTCACCGGGTGTAAATCAGCTTG |

**Table S7. Detail information of protein-protein interactions from the Search Tool for the Retrieval of interacting Genes database (STRING) online.**

| node1 | node2 | neighborhood_on_chromosome | gene_fusion | phylogenetic_cooccurrence | homology | coexpression | experimentally_determined_interaction | database_annotated | automated_textmining | combined_score |
| --- | --- | --- | --- | --- | --- | --- | --- | --- | --- | --- |
| POLR3D | POLR3K | 0 | 0 | 0 | 0 | 0.072 | 0.987 | 0.9 | 0.527 | 0.999 |
| POLR2F | POLR3D | 0 | 0 | 0 | 0 | 0.074 | 0.987 | 0.9 | 0.552 | 0.999 |
| DFFB | DFFA | 0 | 0 | 0 | 0 | 0 | 0.92 | 0.9 | 0.891 | 0.999 |
| CALR | HSP90B1 | 0 | 0 | 0 | 0 | 0.734 | 0.784 | 0.9 | 0.788 | 0.998 |
| APAF1 | CYCS | 0 | 0 | 0 | 0 | 0.055 | 0.36 | 0.9 | 0.961 | 0.997 |
| POLR2F | POLR3K | 0 | 0 | 0 | 0 | 0.206 | 0.923 | 0.9 | 0.584 | 0.997 |
| ACTB | ACTG1 | 0 | 0 | 0 | 0.99 | 0.136 | 0.97 | 0.9 | 0.687 | 0.997 |
| UMPS | DHODH | 0.505 | 0 | 0 | 0 | 0.484 | 0.181 | 0.951 | 0.711 | 0.996 |
| CKS1B | CCNB1 | 0 | 0 | 0 | 0 | 0.697 | 0.962 | 0 | 0.661 | 0.995 |
| VPS36 | TSG101 | 0 | 0 | 0 | 0 | 0.056 | 0.67 | 0.9 | 0.821 | 0.993 |
| POLR2F | POLR2J3 | 0 | 0 | 0 | 0 | 0.667 | 0.844 | 0.54 | 0.724 | 0.992 |
| JUN | TP53 | 0 | 0 | 0 | 0 | 0 | 0.13 | 0.9 | 0.919 | 0.992 |
| TP53 | CCNB1 | 0 | 0 | 0 | 0 | 0.113 | 0.102 | 0.9 | 0.878 | 0.989 |
| JUN | CCND1 | 0 | 0 | 0 | 0 | 0 | 0 | 0.9 | 0.88 | 0.987 |
| APAF1 | XIAP | 0 | 0 | 0 | 0 | 0.127 | 0.418 | 0.9 | 0.746 | 0.985 |
| APAF1 | TP53 | 0 | 0 | 0 | 0 | 0 | 0 | 0.9 | 0.854 | 0.984 |
| IKBKG | CASP8 | 0 | 0 | 0 | 0 | 0 | 0.326 | 0.9 | 0.683 | 0.976 |
| APAF1 | CASP8 | 0 | 0 | 0 | 0 | 0.178 | 0.656 | 0 | 0.922 | 0.976 |
| NEDD4L | SMAD2 | 0 | 0 | 0 | 0 | 0.052 | 0.678 | 0.9 | 0.294 | 0.975 |
| ATF6 | HSP90B1 | 0 | 0 | 0 | 0 | 0.047 | 0 | 0.9 | 0.734 | 0.972 |
| TUBB2A | ACTB | 0 | 0 | 0 | 0 | 0.124 | 0.17 | 0.9 | 0.651 | 0.971 |
| TP53 | CCND1 | 0 | 0 | 0 | 0 | 0 | 0.36 | 0 | 0.955 | 0.97 |
| ACTB | RAC1 | 0 | 0 | 0 | 0 | 0.102 | 0.328 | 0.9 | 0.53 | 0.967 |
| ATF6 | CALR | 0 | 0 | 0 | 0 | 0 | 0 | 0.9 | 0.656 | 0.964 |
| TP53 | SESN2 | 0 | 0 | 0 | 0 | 0 | 0 | 0.9 | 0.634 | 0.961 |
| PARD6B | RAC1 | 0 | 0 | 0 | 0 | 0.085 | 0.685 | 0.8 | 0.404 | 0.961 |
| NEDD4L | TSG101 | 0 | 0 | 0 | 0 | 0.081 | 0 | 0.9 | 0.612 | 0.961 |
| NME2 | RRM2 | 0 | 0 | 0 | 0 | 0.167 | 0 | 0.941 | 0.157 | 0.955 |
| JUN | ACTB | 0 | 0 | 0 | 0 | 0.049 | 0.138 | 0.9 | 0.498 | 0.953 |
| RAC1 | ACTG1 | 0 | 0 | 0 | 0 | 0.054 | 0.1 | 0.9 | 0.508 | 0.952 |
| TP53 | EIF2AK2 | 0 | 0 | 0 | 0 | 0.051 | 0.36 | 0.9 | 0.298 | 0.951 |
| NME1-NME2 | RRM2 | 0 | 0 | 0 | 0 | 0.12 | 0 | 0.941 | 0.147 | 0.951 |
| HSPA4L | HSPA2 | 0 | 0 | 0.49 | 0.779 | 0.291 | 0.12 | 0.9 | 0.529 | 0.946 |
| IKBKG | XIAP | 0 | 0 | 0 | 0 | 0.122 | 0 | 0.9 | 0.41 | 0.943 |
| CASP8 | TP53 | 0 | 0 | 0 | 0 | 0.103 | 0.329 | 0 | 0.91 | 0.941 |
| ADAR | STAT2 | 0 | 0 | 0 | 0 | 0.23 | 0 | 0.9 | 0.29 | 0.94 |
| RRM2 | CCNB1 | 0 | 0 | 0 | 0 | 0.909 | 0 | 0 | 0.35 | 0.938 |
| PRIM1 | POLD3 | 0 | 0 | 0 | 0 | 0.337 | 0 | 0.9 | 0.111 | 0.935 |
| CCNB1 | CCND1 | 0 | 0 | 0 | 0.65 | 0.087 | 0.071 | 0.9 | 0.848 | 0.934 |
| KPNA2 | CCNB1 | 0 | 0 | 0 | 0 | 0.872 | 0.394 | 0 | 0.221 | 0.934 |
| MAX | TP53 | 0 | 0 | 0 | 0 | 0 | 0 | 0.9 | 0.304 | 0.927 |
| CCND3 | CCNB1 | 0 | 0 | 0 | 0.69 | 0.087 | 0.071 | 0.9 | 0.716 | 0.927 |
| DNAJA1 | HSPA4L | 0.309 | 0 | 0 | 0 | 0.4 | 0.592 | 0.274 | 0.485 | 0.925 |
| CASP8 | XIAP | 0 | 0 | 0 | 0 | 0.093 | 0.545 | 0 | 0.829 | 0.923 |
| MAX | SMAD2 | 0 | 0 | 0 | 0 | 0.089 | 0.103 | 0.9 | 0.158 | 0.922 |
| CASP8 | CYCS | 0 | 0 | 0 | 0 | 0 | 0 | 0 | 0.922 | 0.922 |
| POLR2F | NME2 | 0 | 0 | 0 | 0 | 0.189 | 0.069 | 0.9 | 0.051 | 0.918 |
| NME2 | POLR3K | 0 | 0 | 0 | 0 | 0.108 | 0.098 | 0.9 | 0.09 | 0.917 |
| CASP8 | ITGB3 | 0 | 0 | 0 | 0 | 0 | 0 | 0.9 | 0.176 | 0.914 |
| RAC1 | CCND1 | 0 | 0 | 0 | 0 | 0.051 | 0.099 | 0.9 | 0.103 | 0.913 |
| MAX | CCNB1 | 0 | 0 | 0 | 0 | 0 | 0 | 0.9 | 0.152 | 0.911 |
| DNAJA1 | HSPA2 | 0.309 | 0 | 0.374 | 0 | 0.4 | 0.329 | 0.274 | 0.423 | 0.91 |
| NME1-NME2 | POLR3K | 0 | 0 | 0 | 0 | 0.042 | 0.098 | 0.9 | 0.09 | 0.91 |
| POLR2F | NME1-NME2 | 0 | 0 | 0 | 0 | 0.093 | 0.069 | 0.9 | 0.051 | 0.909 |
| DNAJA1 | HSPA1B | 0.309 | 0 | 0.363 | 0 | 0.4 | 0.329 | 0.274 | 0.425 | 0.908 |
| CCND3 | CCND1 | 0 | 0 | 0 | 0.952 | 0.045 | 0 | 0.9 | 0.821 | 0.904 |
| NME1-NME2 | PRIM1 | 0 | 0 | 0 | 0 | 0.073 | 0 | 0.9 | 0 | 0.903 |
| NME2 | MAX | 0 | 0 | 0 | 0 | 0 | 0 | 0.9 | 0.074 | 0.903 |
| POLR2F | JUN | 0 | 0 | 0 | 0 | 0 | 0 | 0.9 | 0.075 | 0.903 |
| NME2 | PRIM1 | 0 | 0 | 0 | 0 | 0.073 | 0 | 0.9 | 0 | 0.903 |
| NME1-NME2 | POLR2J3 | 0 | 0 | 0 | 0 | 0.055 | 0 | 0.9 | 0.05 | 0.902 |
| POLR2F | ACTB | 0 | 0 | 0 | 0 | 0.053 | 0 | 0.9 | 0.051 | 0.902 |
| NME2 | POLR2J3 | 0 | 0 | 0 | 0 | 0.055 | 0 | 0.9 | 0.05 | 0.902 |
| NME1-NME2 | POLR3D | 0 | 0 | 0 | 0 | 0.053 | 0 | 0.9 | 0 | 0.901 |
| NME2 | POLR3D | 0 | 0 | 0 | 0 | 0.053 | 0 | 0.9 | 0 | 0.901 |
| NME2 | POLD3 | 0 | 0 | 0 | 0 | 0 | 0 | 0.9 | 0.046 | 0.9 |
| POLR2F | POLD3 | 0 | 0 | 0 | 0 | 0 | 0 | 0.9 | 0 | 0.9 |
| NME1-NME2 | POLD3 | 0 | 0 | 0 | 0 | 0 | 0 | 0.9 | 0.046 | 0.9 |
| MAX | POLR3D | 0 | 0 | 0 | 0 | 0 | 0 | 0.9 | 0.048 | 0.9 |
| POLR2F | HNRNPUL1 | 0 | 0 | 0 | 0 | 0 | 0 | 0.9 | 0 | 0.9 |
| NME2 | NME1-NME2 | 0 | 0 | 0.527 | 0.989 | 0 | 0.866 | 0 | 0.748 | 0.868 |
| MSN | ACTB | 0 | 0 | 0 | 0 | 0.053 | 0 | 0.8 | 0.336 | 0.863 |
| PPM1D | TP53 | 0 | 0 | 0 | 0 | 0 | 0.326 | 0 | 0.779 | 0.845 |
| RRM2 | PRIM1 | 0 | 0 | 0 | 0 | 0.803 | 0 | 0 | 0.244 | 0.845 |
| HSP90B1 | HSPA2 | 0 | 0 | 0 | 0 | 0.41 | 0.421 | 0 | 0.568 | 0.839 |
| JUN | CYCS | 0 | 0 | 0 | 0 | 0 | 0 | 0 | 0.837 | 0.837 |
| MSN | ACTG1 | 0 | 0 | 0 | 0 | 0.053 | 0 | 0.8 | 0.195 | 0.834 |
| HSPA1B | HSP90B1 | 0 | 0 | 0 | 0 | 0.41 | 0.421 | 0 | 0.547 | 0.831 |
| CYCS | TP53 | 0 | 0 | 0 | 0 | 0 | 0 | 0 | 0.826 | 0.826 |
| NEDD4L | PDGFRA | 0 | 0 | 0 | 0 | 0.059 | 0.12 | 0.8 | 0.069 | 0.825 |
| DNAJA1 | HSP90B1 | 0 | 0 | 0 | 0 | 0.47 | 0.325 | 0 | 0.538 | 0.82 |
| IKBKG | CCND1 | 0 | 0 | 0 | 0 | 0 | 0 | 0.72 | 0.344 | 0.808 |
| HSP90B1 | HSPA4L | 0 | 0 | 0 | 0 | 0.368 | 0.421 | 0 | 0.487 | 0.796 |
| ACTB | TP53 | 0 | 0 | 0 | 0 | 0.09 | 0 | 0 | 0.776 | 0.788 |
| POLR2J3 | POLR3K | 0.309 | 0 | 0 | 0 | 0.269 | 0.534 | 0 | 0.159 | 0.775 |
| RRM2 | CKS1B | 0 | 0 | 0 | 0 | 0.734 | 0 | 0 | 0.168 | 0.769 |
| JUN | SMAD2 | 0 | 0 | 0 | 0 | 0 | 0.37 | 0 | 0.642 | 0.765 |
| XIAP | CYCS | 0 | 0 | 0 | 0 | 0 | 0 | 0 | 0.754 | 0.754 |
| CCND3 | IKBKG | 0 | 0 | 0 | 0 | 0 | 0 | 0.72 | 0.11 | 0.74 |
| RRM2 | TP53 | 0 | 0 | 0 | 0 | 0.088 | 0.285 | 0 | 0.631 | 0.739 |
| TUBB2A | ACTG1 | 0 | 0 | 0 | 0 | 0.124 | 0.17 | 0 | 0.669 | 0.738 |
| TP53 | SMAD2 | 0 | 0 | 0 | 0 | 0.049 | 0.387 | 0 | 0.574 | 0.73 |
| JUN | CASP8 | 0 | 0 | 0 | 0 | 0 | 0.067 | 0 | 0.708 | 0.716 |
| XIAP | TP53 | 0 | 0 | 0 | 0 | 0 | 0 | 0 | 0.708 | 0.708 |
| PRIM1 | CKS1B | 0 | 0 | 0 | 0 | 0.686 | 0 | 0 | 0.092 | 0.704 |
| KPNA2 | CKS1B | 0 | 0 | 0 | 0 | 0.516 | 0.295 | 0 | 0.189 | 0.699 |
| RRM2 | KPNA2 | 0 | 0 | 0 | 0 | 0.646 | 0.071 | 0 | 0.14 | 0.692 |
| KPNA2 | TP53 | 0 | 0 | 0 | 0 | 0.068 | 0.36 | 0 | 0.516 | 0.686 |
| PRIM1 | CCNB1 | 0 | 0 | 0 | 0 | 0.618 | 0 | 0 | 0.201 | 0.682 |
| NME2 | CCND1 | 0 | 0 | 0 | 0 | 0 | 0.068 | 0 | 0.664 | 0.673 |
| IRAK4 | IKBKG | 0 | 0 | 0 | 0 | 0 | 0.397 | 0 | 0.475 | 0.67 |
| CKS1B | TP53 | 0 | 0 | 0 | 0 | 0.075 | 0 | 0 | 0.65 | 0.662 |
| NME1-NME2 | CCND1 | 0 | 0 | 0 | 0 | 0 | 0.068 | 0 | 0.641 | 0.651 |
| DFFA | CASP8 | 0 | 0 | 0 | 0 | 0 | 0.054 | 0 | 0.63 | 0.635 |
| ACTB | CYCS | 0 | 0 | 0 | 0 | 0.062 | 0 | 0 | 0.626 | 0.634 |
| APAF1 | DFFA | 0 | 0 | 0 | 0 | 0 | 0 | 0 | 0.629 | 0.629 |
| CCND3 | TP53 | 0 | 0 | 0 | 0 | 0 | 0 | 0 | 0.628 | 0.628 |
| HSP90B1 | TP53 | 0 | 0 | 0 | 0 | 0 | 0.387 | 0 | 0.407 | 0.621 |
| POLR3K | UMPS | 0.056 | 0 | 0 | 0 | 0.229 | 0 | 0 | 0.503 | 0.607 |
| HSPA1B | TP53 | 0 | 0 | 0 | 0 | 0.052 | 0.424 | 0 | 0.334 | 0.605 |
| CIITA | STAT2 | 0 | 0 | 0 | 0 | 0.055 | 0 | 0 | 0.599 | 0.605 |
| POLR2F | UMPS | 0.505 | 0 | 0 | 0 | 0.163 | 0 | 0 | 0.08 | 0.585 |
| TP53 | PDGFRA | 0 | 0 | 0 | 0 | 0 | 0.063 | 0 | 0.573 | 0.582 |
| CKS1B | CCND1 | 0 | 0 | 0 | 0 | 0.054 | 0.296 | 0 | 0.419 | 0.579 |
| CYCS | CCND1 | 0 | 0 | 0 | 0 | 0 | 0 | 0 | 0.577 | 0.577 |
| DFFA | CYCS | 0 | 0 | 0 | 0 | 0 | 0 | 0 | 0.577 | 0.577 |
| CASP8 | CCND1 | 0 | 0 | 0 | 0 | 0 | 0 | 0 | 0.576 | 0.576 |
| PPM1D | PDGFRA | 0.309 | 0 | 0 | 0 | 0.092 | 0.169 | 0 | 0.283 | 0.576 |
| POLR2J3 | POLR3D | 0 | 0 | 0 | 0 | 0.073 | 0.55 | 0 | 0.065 | 0.575 |
| NME2 | UMPS | 0.062 | 0 | 0 | 0 | 0.092 | 0 | 0 | 0.525 | 0.56 |
| IRAK4 | PPM1D | 0.309 | 0 | 0 | 0 | 0.092 | 0.169 | 0 | 0.254 | 0.558 |
| TP53 | TSG101 | 0 | 0 | 0 | 0 | 0 | 0.36 | 0 | 0.337 | 0.557 |
| HSPA4L | UMPS | 0.08 | 0 | 0 | 0 | 0.097 | 0 | 0 | 0.505 | 0.552 |
| HSPA2 | UMPS | 0.08 | 0 | 0 | 0 | 0.097 | 0 | 0 | 0.505 | 0.552 |
| HSPA1B | UMPS | 0.08 | 0 | 0 | 0 | 0.097 | 0 | 0 | 0.505 | 0.552 |
| APAF1 | JUN | 0 | 0 | 0 | 0 | 0 | 0 | 0 | 0.55 | 0.55 |
| NME2 | TP53 | 0 | 0 | 0 | 0 | 0.076 | 0 | 0 | 0.531 | 0.548 |
| IRAK4 | UMPS | 0 | 0 | 0 | 0 | 0 | 0.372 | 0 | 0.31 | 0.548 |
| PDGFRA | UMPS | 0 | 0 | 0 | 0 | 0 | 0.372 | 0 | 0.31 | 0.548 |
| JUN | XIAP | 0 | 0 | 0 | 0 | 0.053 | 0 | 0 | 0.542 | 0.548 |
| ACTB | HSP90B1 | 0 | 0 | 0 | 0 | 0.063 | 0.054 | 0 | 0.521 | 0.539 |
| MSN | CALR | 0 | 0 | 0 | 0 | 0.051 | 0.424 | 0 | 0.214 | 0.533 |
| XIAP | CCND1 | 0 | 0 | 0 | 0 | 0.045 | 0 | 0 | 0.531 | 0.532 |
| ACTB | UMPS | 0 | 0 | 0 | 0 | 0.057 | 0 | 0 | 0.523 | 0.531 |
| ACTB | CCNB1 | 0 | 0 | 0 | 0 | 0.052 | 0.096 | 0 | 0.496 | 0.53 |
| NME1-NME2 | UMPS | 0.062 | 0 | 0 | 0 | 0.068 | 0 | 0 | 0.505 | 0.529 |
| APAF1 | HSP90B1 | 0 | 0 | 0 | 0 | 0 | 0.409 | 0 | 0.231 | 0.527 |
| JUN | STAT2 | 0 | 0 | 0 | 0 | 0.052 | 0.17 | 0 | 0.432 | 0.514 |
| ACTG1 | UMPS | 0 | 0 | 0 | 0 | 0.057 | 0 | 0 | 0.504 | 0.512 |
| DFFB | CASP8 | 0 | 0 | 0 | 0 | 0.069 | 0.104 | 0 | 0.458 | 0.509 |
| CCND3 | CKS1B | 0 | 0 | 0 | 0 | 0.054 | 0.296 | 0 | 0.322 | 0.509 |
| RAC1 | XIAP | 0 | 0 | 0 | 0 | 0 | 0.508 | 0 | 0 | 0.509 |
| JUN | CCNB1 | 0 | 0 | 0 | 0 | 0 | 0.067 | 0 | 0.492 | 0.506 |
| CASP8 | EIF2AK2 | 0 | 0 | 0 | 0 | 0.053 | 0.36 | 0 | 0.247 | 0.504 |
| ACTG1 | HSP90B1 | 0 | 0 | 0 | 0 | 0.063 | 0.054 | 0 | 0.476 | 0.494 |
| DNAJA1 | UMPS | 0.107 | 0 | 0 | 0 | 0.051 | 0 | 0 | 0.45 | 0.493 |
| JUN | IKBKG | 0 | 0 | 0 | 0 | 0 | 0 | 0 | 0.491 | 0.491 |
| APAF1 | DFFB | 0 | 0 | 0 | 0 | 0 | 0 | 0 | 0.487 | 0.487 |
| TUBB2A | CCNB1 | 0 | 0 | 0 | 0 | 0.085 | 0.106 | 0 | 0.421 | 0.485 |
| CASP8 | CCNB1 | 0 | 0 | 0 | 0 | 0.05 | 0 | 0 | 0.479 | 0.484 |
| CYCS | CCNB1 | 0 | 0 | 0 | 0 | 0 | 0 | 0 | 0.481 | 0.481 |
| ATF6 | EIF2AK2 | 0 | 0 | 0 | 0 | 0.053 | 0 | 0 | 0.475 | 0.481 |
| ACTG1 | CYCS | 0 | 0 | 0 | 0 | 0.062 | 0 | 0 | 0.469 | 0.48 |
| STAT2 | EIF2AK2 | 0 | 0 | 0 | 0 | 0.211 | 0 | 0 | 0.368 | 0.48 |
| TUBB2A | RAC1 | 0 | 0 | 0 | 0 | 0.057 | 0.293 | 0 | 0.282 | 0.479 |
| HSPA1B | HSPA4L | 0 | 0 | 0.486 | 0.796 | 0.268 | 0.12 | 0 | 0.603 | 0.464 |
| HNRNPUL1 | TP53 | 0 | 0 | 0 | 0 | 0 | 0.36 | 0 | 0.194 | 0.462 |
| MSN | HSPA4L | 0 | 0 | 0 | 0 | 0.061 | 0.444 | 0 | 0.052 | 0.462 |
| ATXN3 | DNAJA1 | 0 | 0 | 0 | 0 | 0.044 | 0.173 | 0 | 0.367 | 0.455 |
| CYCS | UMPS | 0 | 0 | 0 | 0 | 0.118 | 0 | 0 | 0.404 | 0.452 |
| SMAD2 | CCND1 | 0 | 0 | 0 | 0 | 0.054 | 0 | 0 | 0.443 | 0.451 |
| JUN | UMPS | 0 | 0 | 0 | 0 | 0 | 0.067 | 0 | 0.428 | 0.444 |
| JUN | ATF6 | 0 | 0 | 0 | 0 | 0 | 0 | 0 | 0.443 | 0.443 |
| IKBKG | TP53 | 0 | 0 | 0 | 0 | 0 | 0 | 0 | 0.443 | 0.443 |
| RAC1 | KPNA2 | 0 | 0 | 0 | 0 | 0.053 | 0.415 | 0 | 0.074 | 0.442 |
| JUN | TSG101 | 0 | 0 | 0 | 0 | 0 | 0.36 | 0 | 0.163 | 0.442 |
| ITGB3 | PDGFRA | 0 | 0 | 0 | 0 | 0.054 | 0.368 | 0 | 0.138 | 0.441 |
| ATF6 | CYCS | 0 | 0 | 0 | 0 | 0 | 0 | 0 | 0.441 | 0.441 |
| APAF1 | CCND1 | 0 | 0 | 0 | 0 | 0 | 0 | 0 | 0.44 | 0.44 |
| ADAR | EIF2AK2 | 0 | 0 | 0 | 0.615 | 0.272 | 0 | 0 | 0.594 | 0.433 |
| CALR | TP53 | 0 | 0 | 0 | 0 | 0 | 0 | 0 | 0.432 | 0.432 |
| XIAP | CCNB1 | 0 | 0 | 0 | 0 | 0.064 | 0.143 | 0 | 0.348 | 0.431 |
| HSPA1B | ACTB | 0 | 0 | 0 | 0 | 0.055 | 0.044 | 0 | 0.411 | 0.422 |
| ACTB | CCND1 | 0 | 0 | 0 | 0 | 0.049 | 0 | 0 | 0.415 | 0.419 |
| CALR | CYCS | 0 | 0 | 0 | 0 | 0.054 | 0 | 0 | 0.409 | 0.417 |
| DNAJA1 | ACTB | 0 | 0 | 0 | 0 | 0.055 | 0 | 0 | 0.407 | 0.415 |
| PRIM1 | TP53 | 0 | 0 | 0 | 0 | 0 | 0.36 | 0 | 0.12 | 0.413 |
| IRAK4 | CLEC7A | 0 | 0 | 0 | 0 | 0.189 | 0.071 | 0 | 0.277 | 0.408 |
| DFFB | CYCS | 0 | 0 | 0 | 0 | 0 | 0 | 0 | 0.409 | 0.408 |
| ATF6 | TP53 | 0 | 0 | 0 | 0 | 0 | 0 | 0 | 0.409 | 0.408 |
| DFFA | TP53 | 0 | 0 | 0 | 0 | 0 | 0 | 0 | 0.408 | 0.408 |
| NME2 | JUN | 0 | 0 | 0 | 0 | 0 | 0 | 0 | 0.407 | 0.407 |
| CCND3 | JUN | 0 | 0 | 0 | 0 | 0 | 0 | 0 | 0.405 | 0.405 |
| MSN | CASP8 | 0 | 0 | 0 | 0 | 0.054 | 0.324 | 0 | 0.142 | 0.403 |
| APAF1 | HSPA1B | 0 | 0 | 0 | 0 | 0.048 | 0.328 | 0 | 0.141 | 0.403 |
| PRIM1 | KPNA2 | 0 | 0 | 0 | 0 | 0.401 | 0 | 0 | 0 | 0.402 |
| NEDD4L | VPS36 | 0 | 0 | 0 | 0 | 0.062 | 0 | 0 | 0.388 | 0.401 |
| POLR2F | PDGFRA | 0.309 | 0 | 0 | 0 | 0 | 0.071 | 0 | 0.141 | 0.401 |
| ATF6 | CASP8 | 0 | 0 | 0 | 0 | 0.074 | 0 | 0 | 0.38 | 0.401 |


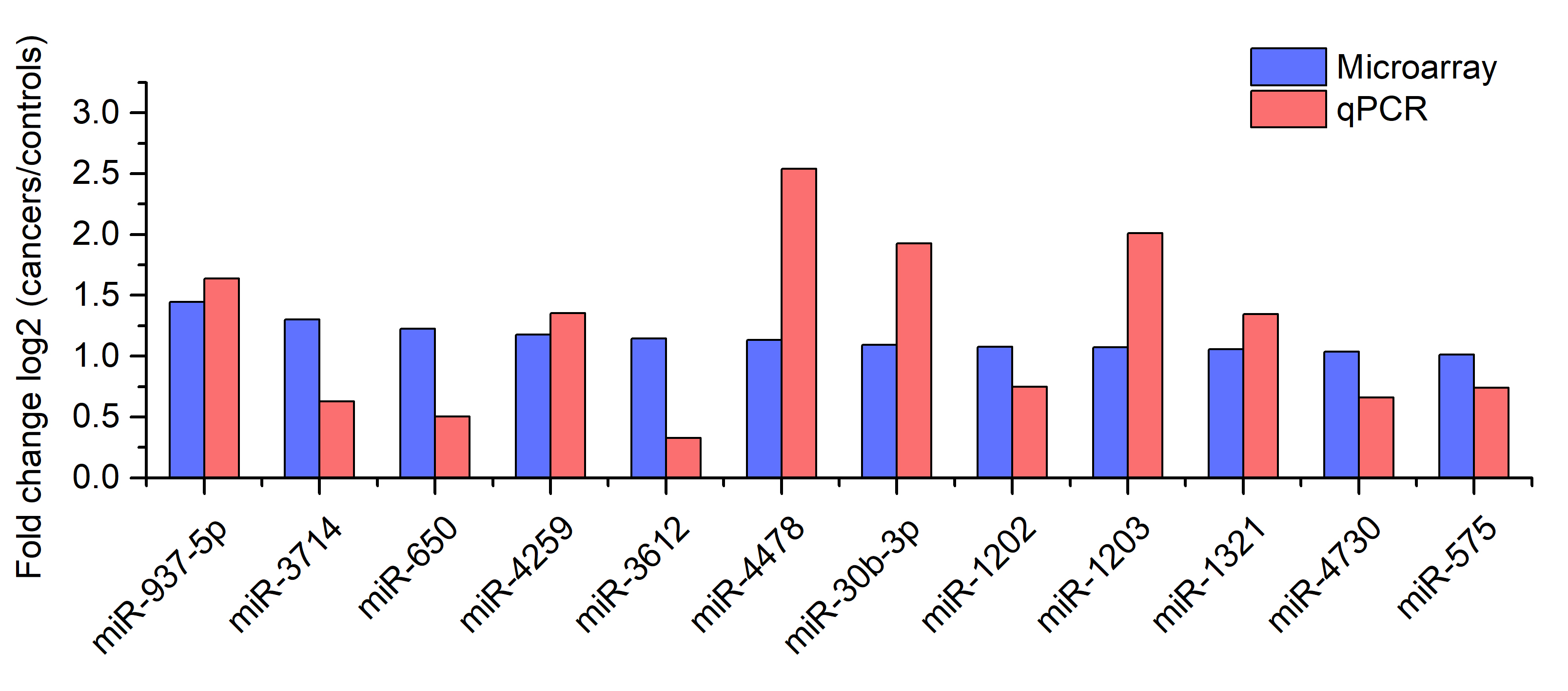


**Figure S1.** Validation of the miRNA expression (miR-937-5p, miR-650, miR-3612, miR-4478, miR-4259, miR-3714, miR-4730, miR-1203, miR-30b-3p, miR-1321, miR-1202, and miR-575) by qRT-PCR in 22 patients and 25 healthy controls.


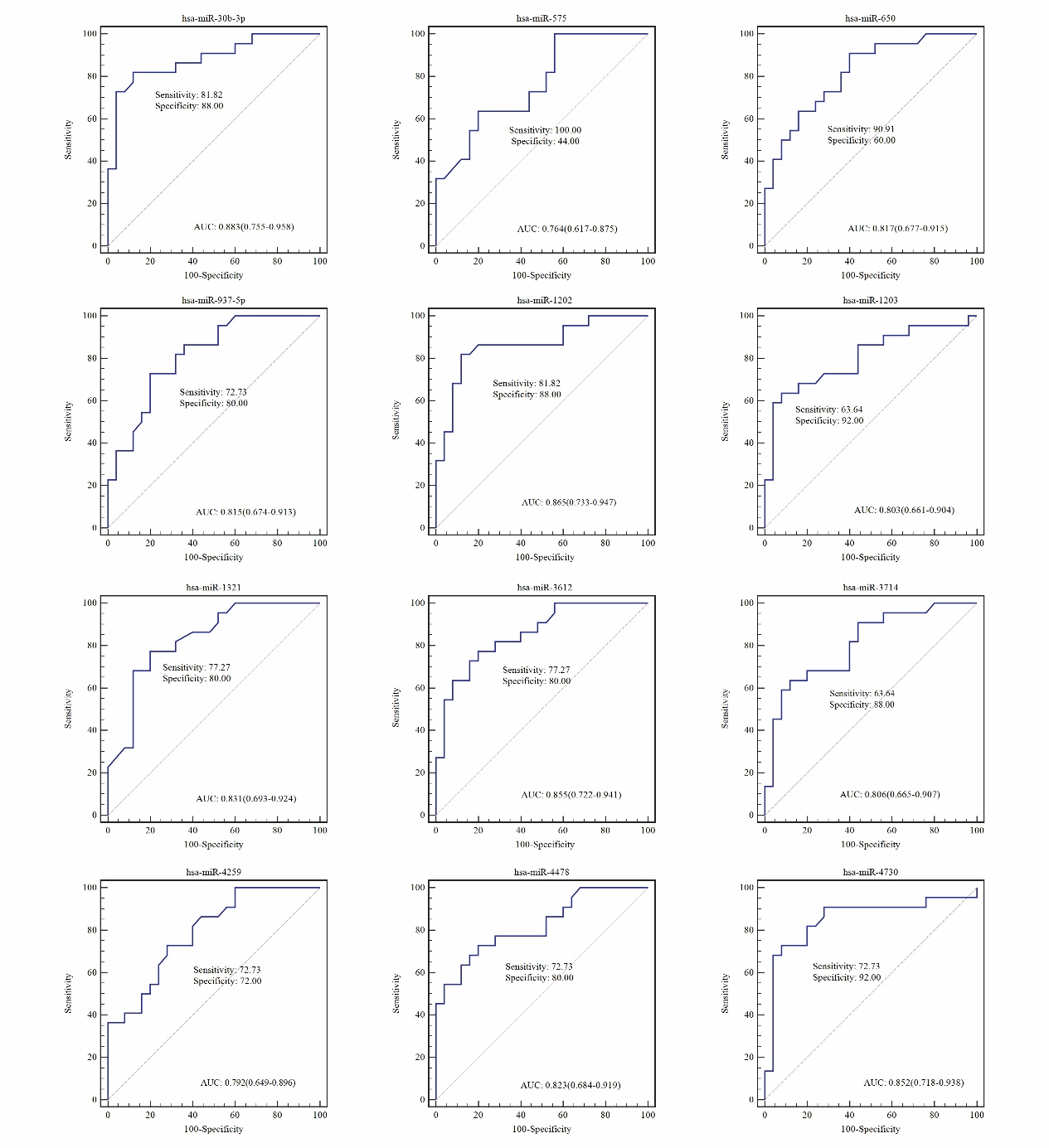


**Figure S2.** ROC curves of the diagnostic potential of the 12 individual salivary miRNAs (has-miR-30b-3p, has-miR-575, has-miR-650, has-miR-937-5p, has-miR-1202, has-miR-1203, has-miR-1321, has-miR-3612, has-miR-3714, has-miR-4259, has-miR-4478, and has-miR-4730) in discrimination between NPC patients and healthy controls. The AUC values ranged from 0.764 to 0.883, respectively.


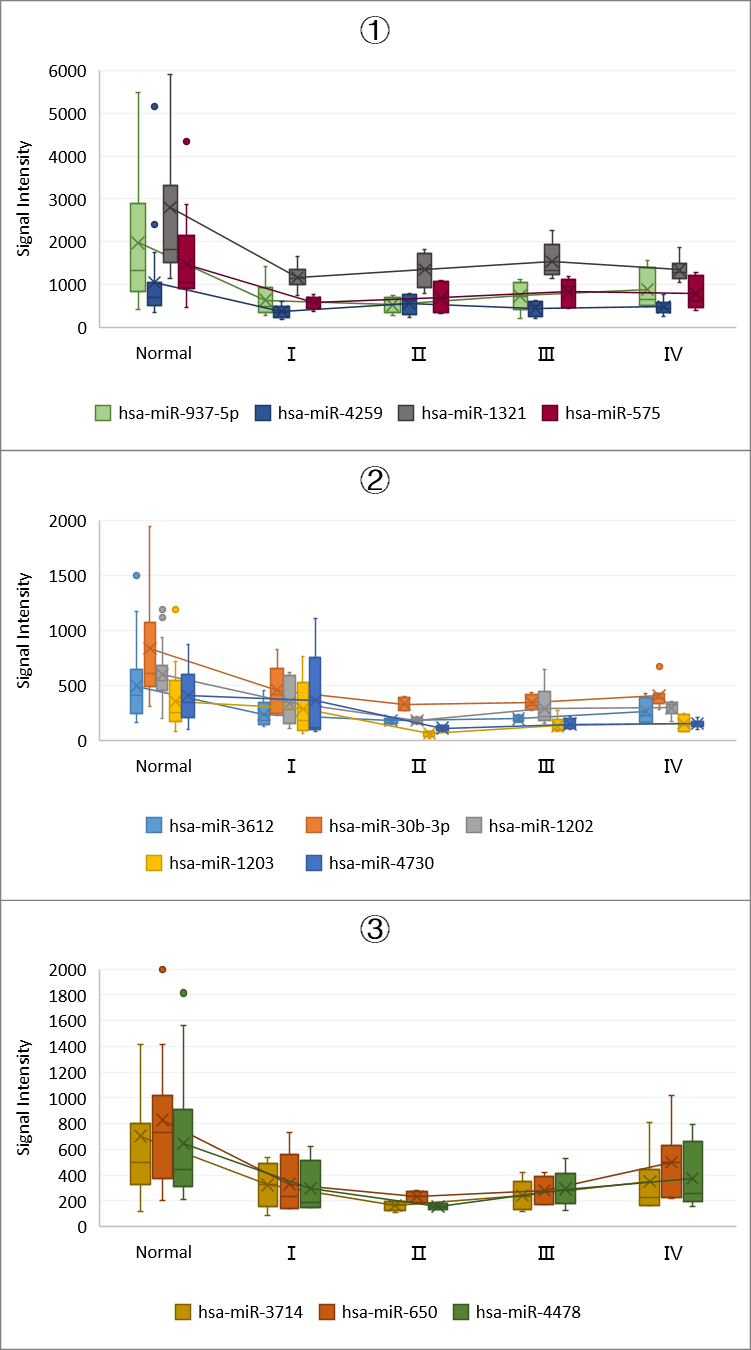


**Figure S3.** Diagnostic miRNA expressions were classified into 3 different patterns based on various clinical stages.
